# Mutations in the spliceosomal gene *SNW1* cause neurodevelopment disorders with microcephaly

**DOI:** 10.1172/JCI186119

**Published:** 2025-07-03

**Authors:** Lei Ji, Jin Yan, Nicole A. Losurdo, Hua Wang, Liangjie Liu, Keyi Li, Zhen Liu, Zhenming Guo, Jing Xu, Adriana Bibo, Decheng Ren, Ke Yang, Yingying Luo, Fengping Yang, Gui Wang, Zhenglong Xiang, Yuan Wang, Huaizhe Zhan, Hu Pan, Juanli Hu, Jianmin Zhong, Rami Abou Jamra, Pia Zacher, Luciana Musante, Flavio Faletra, Paola Costa, Caterina Zanus, Nathalie Couque, Lyse Ruaud, Anna M. Cueto-González, Hector San Nicolas Fernández, Eduardo Tizzano, Nuria Martinez Gil, Xiaorong Liu, Weiping Liao, Layal Abi Farraj, Alden Y. Huang, Liying Zhang, Aparna Murali, Esther Schmuel, Christina S. Han, Kayla King, Weiyue Gu, Pengchao Wang, Kai Li, Nichole Link, Guang He, Shan Bian, Xiao Mao

**Affiliations:** 1 Bio-X Institutes, Key Laboratory for the Genetics of Developmental and Neuropsychiatric Disorders, and; 2 Shanghai Institute of Medical Genetics, Shanghai Children’s Hospital, Shanghai Jiao Tong University, Shanghai, China.; 3 Institute for Regenerative Medicine, State Key Laboratory of Cardiology and Medical Innovation Center, Shanghai East Hospital, Frontier Science Center for Stem Cell Research, School of Life Sciences and Technology, Tongji University, Shanghai, China.; 4 Department of Neurobiology, The University of Utah, Salt Lake City, Utah, USA.; 5 The Affiliated Children’s Hospital of Xiangya School of Medicine, Central South University, Changsha, China.; 6 Clinical Medical Research Center for Hereditary Birth Defects and Rare Diseases in Hunan Province, Changsha, China.; 7 Department of Medical Genetics and; 8 National Health Commission Key Laboratory for Birth Defect Research and Prevention, Hunan Provincial Maternal and Child Health Care Hospital, Changsha, China.; 9 Department of Neurology, Jiangxi Provincial Children’s Hospital, Nanchang, China.; 10 Institute of Human Genetics, University of Leipzig Medical Center, Leipzig, Germany.; 11 Kleinwachau Epilepsy Center, Radeberg, Germany.; 12 Institute for Maternal and Child Health, IRCCS “Burlo Garofolo,” Trieste, Italy.; 13 Institute of Medical Genetics, Azienda Sanitaria Universitaria Friuli Centrale, Udine, Italy.; 14 Department of Medicine, University of Udine, Udine, ltaly.; 15 Department of Genetics, AP-HP–Robert Debré University Hospital, Paris, France.; 16 Laboratoire de Biologie Médicale Multisite SeqOIA, Sorbonne University, Paris, France.; 17 Department of Clinical and Molecular Genetics, Vall d’Hebron Barcelona Hospital Campus, Barcelona, Spain.; 18 Medicine Genetics Group, Vall Hebron Research Institute, Vall d’Hebron Barcelona Hospital Campus, Autonomous University of Barcelona, Barcelona, Spain.; 19 Department of Neurology, Institute of Neuroscience, Key Laboratory of Neurogenetics and Channelopathies of Guangdong Province and the Ministry of Education of China, The Second Affiliated Hospital, Guangzhou Medical University, Guangzhou, China.; 20 Department of Pathology and Laboratory Medicine and; 21 Department of Obstetrics and Gynecology, David Geffen School of Medicine at UCLA, Los Angeles, California, USA.; 22 Comprehensive Maternal-Fetal Medicine Center, Thousand Oaks, California, USA.; 23 Department of Human and Molecular Genetics, VCU Health School of Medicine, Richmond, Virginia, USA.; 24 Department of Human and Molecular Genetics, School of Medicine, Virginia Commonwealth University, Richmond, Virginia, USA.; 25 Chigene (Beijing) Translational Medical Research Center Co. Ltd., Beijing, China.; 26 Department of Neurology and Suzhou Clinical Research Center of Neurological Disease, The Second Affiliated Hospital of Soochow University, Suzhou, China.; 27 China Regional Research Centre, International Centre for Genetic Engineering and Biotechnology, Taizhou, China.

**Keywords:** Genetics, Neuroscience, Embryonic stem cells, Genetic diseases, Neurodevelopment

## Abstract

The spliceosome is a critical cellular machinery responsible for pre-mRNA splicing that is essential for the proper expression of genes. Mutations in its core components are increasingly linked to neurodevelopmental disorders, such as primary microcephaly. Here, we investigated the role of SNW domain–containing protein 1 (SNW1), a spliceosomal protein, in splicing integrity and neurodevelopment. We identified 9 heterozygous mutations in the *SNW1* gene in patients presenting with primary microcephaly. These mutations impaired SNW1’s interactions with core spliceosomal proteins, leading to defective RNA splicing and reduced protein functionality. Using *Drosophila melanogaster* and human embryonic stem cell–derived cerebral organoids models, we demonstrated that *SNW1* depletion resulted in significant reductions in neural stem cell proliferation and increased apoptosis. RNA-Seq revealed disrupted alternative splicing, especially skipping exons, and altered expression of neurodevelopment-associated genes (*CENPE*, *MEF2C*, and *NRXN2*). Our findings provide crucial insights into the molecular mechanisms by which *SNW1* dysfunction contributes to neurodevelopmental disorders and underscore the importance of proper spliceosome function in brain development.

## Abstract 2

Ji et al. have investigated mutations in the *SNW1* gene and concluded that these mutations disrupt RNA splicing, leading to impaired neural development.

## Introduction

Mutations in spliceosome components — a key cellular machinery for pre-mRNA splicing — are increasingly implicated in neurodevelopmental disorders (NDDs), notably primary microcephaly (1, 2). The spliceosome, composed of 5 small nuclear RNAs (snRNAs) and approximately 100 proteins, removes introns and joins exons to generate mature mRNA (3, 4). Beyond snRNAs, non–small nuclear ribonucleoprotein factors including the NineTeen complex (NTC) and NTC-related (NTR) proteins are essential for spliceosome activation and catalysis (5–8). Recent electron microscopy studies have resolved the spliceosome’s structure at near-atomic resolution, shedding light on splicing dynamics (9). Pathogenic mutations have been identified in core spliceosomal proteins, including the exon junction complex (EJC) (10), *EFTUD2* (11, 12), *WBP4* (13), *U2AF2*, *PRP19* (2), *PPIL1*, and *PRP17* (14–16). These mutations disrupt alternative splicing (AS), which is especially conserved and functionally critical in the brain (17, 18). The high energy demand of the brain further exacerbates the impact of spliceosomal dysfunction during development (2, 19).

SNW domain–containing protein 1 (SNW1; formerly SKIP) is a highly conserved 536–amino acid spliceosomal protein encoded by a 14-exon gene on chromosome 14, with 2 known isoforms (20). Initially identified in *Drosophila melanogaster*, SNW1 harbors a conserved SNW/SKIP domain with the S-N-W-K-N amino acid sequence and comprises 3 main regions: the N-terminus (residues 1–173), the SNW domain (residues 174–232), and the C-terminus (residues 333–536) (21–23). It functions as a transcriptional coactivator in the Notch signaling (24, 25), TGF-β (26), and vitamin D receptor pathways (27–29).

SNW1 acts as a scaffold protein, facilitating interactions between various spliceosomal components (30, 31). It is involved in the transition from 1 conformational state of the spliceosome to another, particularly during the assembly of the catalytic core. When analyzing the protein-protein, protein-RNA, and RNA-RNA interactions within different spliceosomal structures using network theory, *SNW1* displayed high betweenness centrality (32). This suggests that *SNW1* may serve as a regulatory node during spliceosome activation, bridging the retention and splicing heterotrimeric (RES) and NTR complexes, indicating a potential regulatory function in splicing activation and later stages. *SNW1* is an elongated (nonglobular) protein, which allows it to maintain a highly flexible and dynamic state, playing a significant role in network architecture (32, 33). SNW1’s interactions with other proteins and RNA elements help stabilize the spliceosome’s structure and contribute to its dynamic behavior, influencing the overall architecture and function of the complex (30). Interestingly, SNW1’s close interaction partner, PPIL1, a member of the NTC, has been increasingly associated with microcephaly-related mutations in recent years (14–16). Here, we report that mutations in *SNW1* impair interactions with core spliceosomal members, disrupt RNA splicing integrity, and result in microcephaly phenotypes in both humans and *Drosophila*. Our study aims to elucidate the impact of *SNW1* mutations on protein function and their role in the pathogenesis of primary microcephaly. By exploring the molecular mechanisms underlying these mutations, we hope to contribute to a better understanding of the critical role of the spliceosome in brain development and NDDs.

## Results

### Identification of mutations in SNW1.

**Figure 1:**
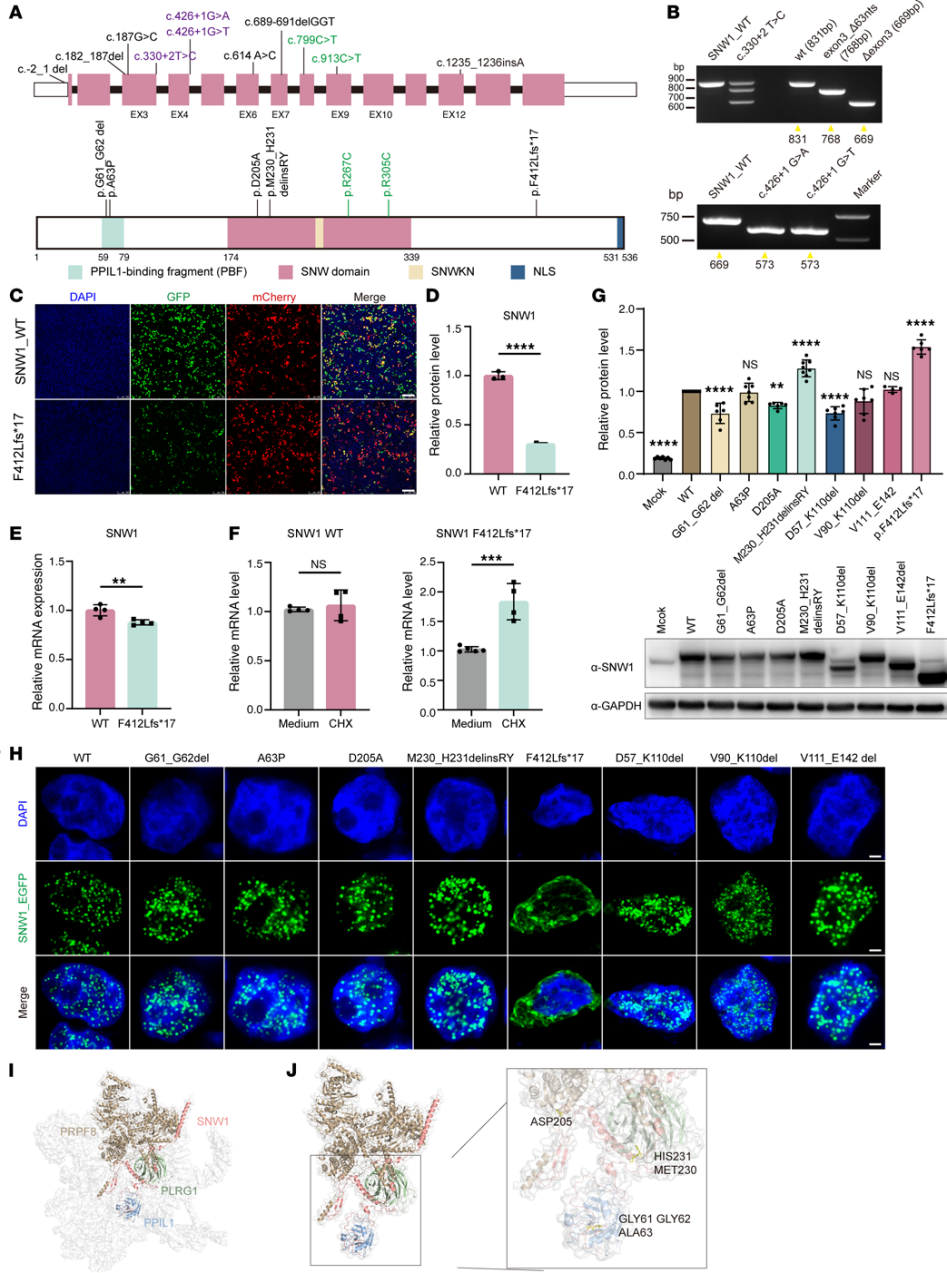
Mutations in *SNW1* lead to microcephaly and impair SNW1 functions in human. (**A**) Schematic diagram of the *SNW1* transcript (NM_012245.3, intron not to scale) (top panel) and schematic outline of the SNW1 protein domains (lower panel) with the locations of 9 loss-of-function variants identified in our study. The splice donor variants are shown in purple. The 2 variants reported in the literature are shown in green. (**B**) The c.330+2T>C construct showed complete skipping of exon 3 and a partial 63-bp skip. The c.426+1G>A and c.426+1G>T constructs showed complete skipping of exon 4. (**C**) Fluorescence images of the HEK293T cells after transfection with SNW1_WT or F412Lfs*17 vectors. pmCherry-C1 was used as an internal control and cotransfected with the WT and F412Lfs*17 vectors at the same ratio. Scale bars: 100 μm. (**D**) Expression analysis of SNW1 by Western blotting was performed in lysates from HEK293T cells transfected with either SNW1 WT or F412Lfs*17 vectors. (**E**) qPCR analysis for SNW1 in HEK293T cells transfected with SNW1 WT or F412Lfs*17 vectors. (**F**) qPCR analysis for SNW1 in SNW1 WT (left) or F412Lfs*17 (right) HEK293T cells after being stimulated by the NMD inhibitor cycloheximide (CHX; 100 μg/mL). (**G**) Overexpression of C-terminal FLAG-tagged WT and SNW1 variants in HEK293T cells. GAPDH served as a loading control. Quantification of overexpressed FLAG-tagged SNW1 proteins. (**H**) Effects of mutations on the localization of SNW1 in HEK293T cells. Fluorescence images were captured using a laser scanning confocal microscope (Leica TCS SP8) with ×63 oil glass. SNW1 (green) and DAPI (blue) are displayed. Scale bars: 2.5 μm. (**I** and **J**) Cryo-electron microscopy structure of the human spliceosome ILS complex (Protein Data Bank ID 6id0) highlighting SNW1 (surface in pink) and its interacting proteins in the spliceosome, including PPIL1 (sky blue), PLRG1 (olive drab), and PRPF8 (brown). Residues in patients were observed to be located at the interface where SNW1 interacts with these proteins, suggesting changes in molecular interactions. Data are presented as mean ± SEM. For comparisons among multiple groups, ordinary 1-way ANOVA was performed followed by Tukey’s multiple-comparison test for post hoc pairwise analysis. For comparisons between 2 independent groups, 2-tailed unpaired Student’s *t* tests were used (without post hoc correction). **P < 0.01, ***P < 0.001, ****P < 0.0001.

From a cohort of 3,699 patients with NDDs, we identified a de novo SNW1 variant in 1 individual with classic primary microcephaly. Using information from GeneMatcher, Chigene (Beijing)’s in-house database, and the China Epilepsy Gene 1.0 project, 9 affected individuals were identified to carry heterozygous variants in *SNW1*, encompassing not only missense variants and microdeletions but also intronic variations (Figure 1A; pathogenic or likely pathogenic variants not directly linked to the clinical phenotype are summarized in Supplemental Table 1; supplemental material available online with this article; https://doi.org/10.1172/JCI186119DS1). Eight variants were confirmed as de novo, while 1 individual’s inheritance could not be tested due to adoption.

Except for individual 7, who underwent medical termination at 19 weeks, all presented with overlapping clinical features (Table 1 and Supplemental Table 2). Moderate-to-profound intellectual disability was present in all 9 patients, and another most prevalent finding was severe microcephaly, which might have been of prenatal onset since 5 patients with available birth medical data showed small head circumference at birth. Seizures were present in 7 individuals, and 1 (individual 3) developed into epileptic encephalopathy. Additional commonly seen neuropsychiatric manifestations include autism and global developmental delay involving speech, cognition, and motor skills. It is worth noting that most individuals, even the fetus, showed obvious dysmorphic facial features, including widely spaced teeth, short nose, triangular face, short face, marked prognathism, and large ears. Brain MRI results indicated corpus callosum hypoplasia in 4 individuals and Dandy-Walker malformation in another 2. Beyond our study, 2 previously reported variants within the SNW domain have been linked to NDDs, reinforcing SNW1’s relevance (34). These data reveal a neurodevelopmental syndrome associated with *SNW1* de novo variants, with core features of severe intellectual disability, microcephaly, brain malformations, and facial dysmorphisms.

### Patient mutations affect SNW1 function.

Among the 9 variants identified, 3 were located at canonical 5′ splice donor sites: c.330+2T>C, c.426+1G>A, and c.426+1G>T (Supplemental Figure 1A). These sites, following exons 3 and 4, conform to the conserved GT dinucleotide rule (Supplemental Figure 1B). Multiple in silico splice prediction tools uniformly indicated that all 3 mutations substantially weaken or abolish the corresponding splice donor sites, likely resulting in exon skipping (Supplemental Table 3), which was validated by minigene splicing assay using the pCAS2 vector (Supplemental Figure 1C). The c.330+2T>C variant generated the WT transcript (831 bp) and 2 aberrant splicing isoforms: 1 lacking the entire exon 3 (768 bp) and another deleting its last 63 bp (669 bp) (Figure 1B and Supplemental Figure 1D). In contrast, both c.426+1G>A and c.426+1G>T resulted in complete skipping of exon 4 (Figure 1B and Supplemental Figure 1E). Since these losses are multiples of 3, the resulting protein products were identified as D57_K110del, V90_K110del, and V111_E142 del.

The single frameshift variant identified, c.1235_1236insA (F412Lfs17), located in exon 12, introduces a premature termination codon in exon 13, predicted to activate the nonsense-mediated mRNA decay (NMD) pathway (Supplemental Figure 1F). To experimentally test this hypothesis, we constructed WT and F412Lfs17 mutant vectors encoding SNW1 as a GFP N-terminal fusion protein and transfected them into HEK293T cells (Supplemental Figure 1G). Cells transfected with *SNW1*-mutated vectors showed reduced fluorescence and protein levels (Figure 1, C and D, and Supplemental Figure 1H). Compared with that in SNW1 WT cells, SNW1 mRNA expression in the mutated vector–transfected cells was reduced (Figure 1E). Following cycloheximide treatment to inhibit NMD, SNW1 mRNA levels remained unchanged in WT-transfected cells but were significantly elevated in mutant-transfected cells (Figure 1F). These findings demonstrated that c.1235_1236insA (F412Lfs17) triggers NMD-mediated degradation of the mutant SNW1 mRNA, thereby inhibiting the expression of truncated protein.

To assess the potential functional impact of exon variant–induced residue alterations, we performed a protein sequence alignment of SNW1 orthologs across various species. Protein sequence alignments of *SNW1* revealed that the mutated residues, including G61, G62, A63, D205, and H231, are strictly conserved across multiple species, suggesting that they may play a crucial role in maintaining protein function and evolution (Supplemental Figure 1I). In addition, all amino acid–altering variants were predicted to be intolerant to variation according to MetaDome analysis (Supplemental Figure 1J). To investigate the impact of patient-derived protein variants, we expressed FLAG-tagged mutant proteins in HEK293T cells. The results demonstrated that different *SNW1* variants affect protein expression (Figure 1G). G61_G62del and D57_K110del showed decreased expression, D205A exhibited a slight decrease, while M230_H231delinsRY and F412Lfs*17 showed significantly increased expression. No significant changes were observed for the remaining mutants.

SNW1 localizes to the nucleus in a speckled pattern, with its nuclear localization signal (NLS) mapped to the last 6 C-terminal residues (22, 25, 29, 35–38). For the subcellular localization analysis, the GFP-tagged SNW1 expression plasmid (pEGFP-SNW1) was transfected into HEK293T cells. Confocal microscopy results revealed that the SNW1-EGFP signal was predominantly distributed in a punctate/granular pattern within the nuclei of HEK293T cells (Figure 1H). The majority of the SNW1 mutations did not alter this nuclear localization pattern. In contrast, the F412Lfs*17 SNW1-EGFP mutant was no longer localized to the nucleus but instead displayed a cytosolic distribution, losing its characteristic granular appearance. As a core component of the spliceosome, SNW1 functions continuously from the B complex to the final intron removal (Supplemental Figure 1K). Positioned at the core, SNW1 facilitates the conformational changes of the spliceosome (32), which involves interactions with multiple proteins (Figure 1, I and J). Our mutations were located precisely at these interfaces, prompting us to consider whether the mutation might affect the stable binding of the protein.

### Interactions and functional implications of SNW1 mutations in the spliceosome complex.

SNW1 does not contain a stably folded domain; rather, it is located in the core region of the splicing complex, functioning like a rope that threads through most of the complex (30). Based on the resolved protein complex structures, we identified that several of our mutations are situated in the interaction regions of SNW1 with other spliceosome components, including PPIL1, PLRG1, and PRPF8 (Figure 1, J and K, and Supplemental Figure 1K). We therefore investigated whether these variants disrupt the binding of SNW1 to these proteins. As part of the NTR, SNW1 is incorporated into the spliceosome during the early B^act^ complex stage and recruits PPIL1 at the mature B complex stage, with the interaction persisting through to the intron lariat spliceosome (ILS) complex after exon excision (Supplemental Figure 1K). Notably, an increasing number of reports have linked *PPIL1* mutations to microcephaly phenotypes, such as the failure of PPIL1 p.R131 to associate with SNW1 (14–16). This further underscores the importance of the interaction between SNW1 and PPIL1 in AS and disease.

**Figure 2:**
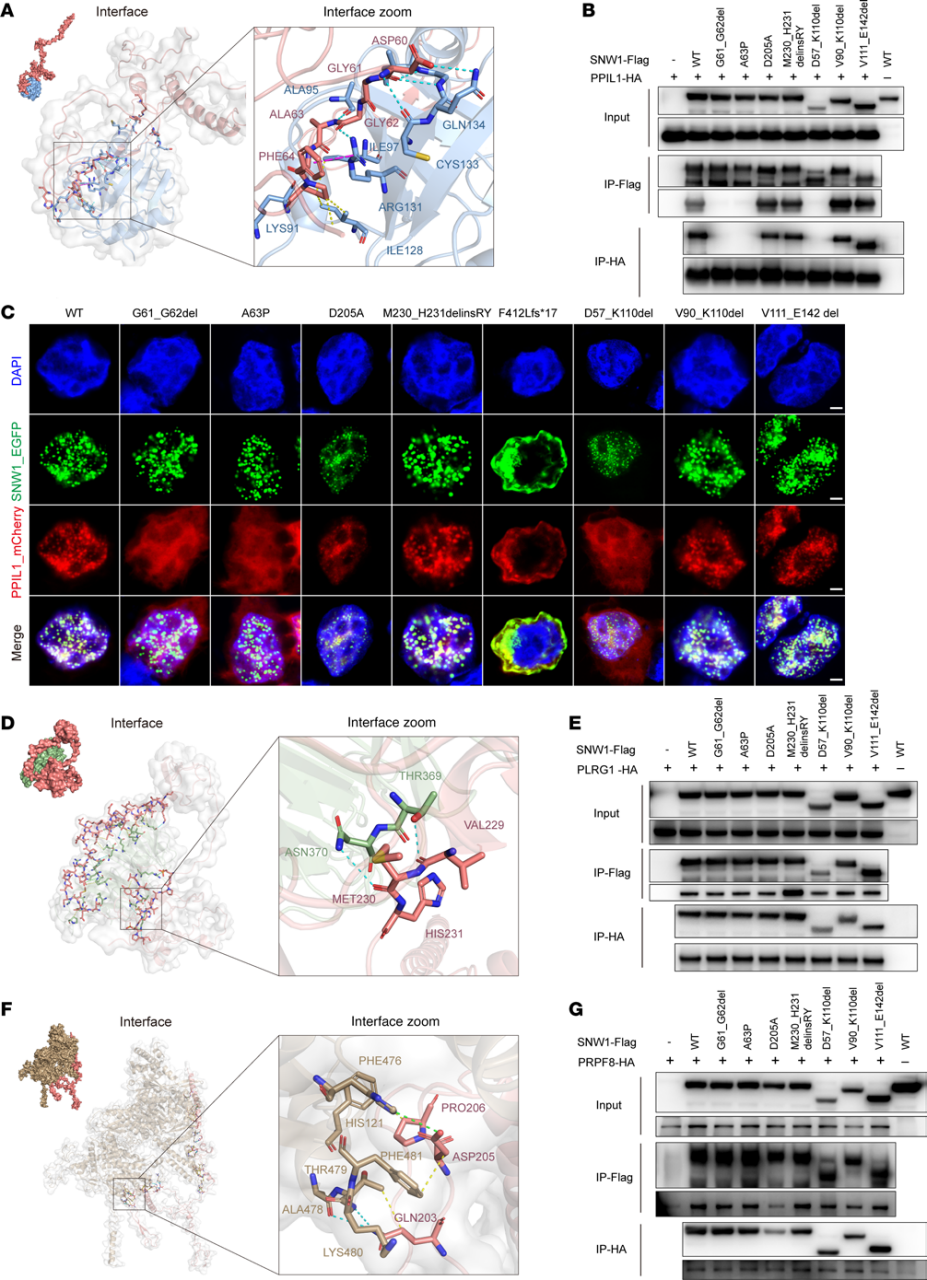
SNW1 interacted with PPIL1, PLRG1, and PRPF8. (**A**) Structure-based protein interaction interface analysis between SNW1 (pink) and PPIL1 (sky blue), where interaction hotspot residues are labeled. (**B**) CoIP of SNW1 and PPIL1 was performed. HEK293T cells were transfected with plasmids encoding SNW1-FLAG and PPIL1-HA. The cell lysates were subjected to anti-FLAG and anti-HA IP, followed by analysis via Western blotting. (**C**) Subcellular localization analysis of SNW1 and PPIL1. HEK293T cells were transfected with the plasmid encoding pEGFP-SNW1 and pmCherry-PPIL1 for 24 hours and then fixed and stained with DAPI. SNW1 (green), PPIL1 (red), and DAPI (blue) are displayed. Scale bars: 2.5 μm. (**D**) Structure-based protein interaction interface analysis between SNW1 (pink) and PLRG1 (olive drab), where interaction hotspot residues are labeled. (**E**) CoIP of SNW1 and PLRG1 was performed. HEK293T cells were transfected with plasmids encoding SNW1-FLAG and PLRG1-HA. The cell lysates were subjected to anti-FLAG and anti-HA IP, followed by analysis via Western blotting. (**F**) Structure-based protein interaction interface analysis between SNW1 (pink) and PRPF8 (brown), with interaction hotspot residues labeled. (**G**) CoIP of SNW1 and PRPF8 was performed. HEK293T cells were transfected with plasmids encoding SNW1-FLAG and PRPF8-HA. The cell lysates were subjected to anti-FLAG and anti-HA IP, followed by analysis via Western blotting.

Studies have shown that SNW1 interacts with PPIL1 via electrostatic and hydrophobic forces involving PPIL1’s β2-α1, β4-β5, and β7 regions and SNW1 residues 59–79 (23, 33, 39, 40). In this study, 3 mutations — G61_G62del, A63P, and D57_K110del (deletion of exon 3) — were located at the PPIL1-binding interface. Specifically, analysis of the ILS complex revealed that SNW1 residues GLY62 and ALA63 form hydrogen bonds with ARG131 and ALA95 of PPIL1, respectively (Figure 2A). Coimmunoprecipitation (CoIP) assays using FLAG-tagged SNW1 mutants and HA-tagged PPIL1 confirmed that these 3 variants lost PPIL1 binding, while others remained unaffected (Figure 2B). To further explore the interaction between SNW1 and PPIL1, we examined their subcellular colocalization in 3 cell lines. PPIL1 alone was distributed evenly in both the nucleus and cytoplasm, with slight nuclear enrichment (Supplemental Figure 2A). However, upon coexpression with SNW1-WT or other binding-competent SNW1 variants, PPIL1 localization was restricted to the nucleus, suggesting that SNW1 recruits PPIL1 to the spliceosome (Figure 2C and Supplemental Figure 2, B and C). In contrast, coexpression with the G61_G62del, A63P, or D57_K110del mutant led to persistent cytoplasmic PPIL1 signals, reflecting their loss of binding. Notably, the F412Lfs*17 mutant retained PPIL1-binding ability but failed to recruit it to the nucleus due to NLS loss, further supporting the regulatory role of SNW1 subcellular localization in determining PPIL1 distribution.

PLRG1, a conserved NTC component, is crucial for alternative splice site selection. In yeast, the interaction between prp45 and prp46 has been confirmed to be essential for pre-mRNA splicing (41). Within the spliceosome, hydrogen bonds are present between SNW1’s VAL229 and MET230 and PLRG1’s THR369 and ASN370 (Figure 2D). CoIP results indicated that M230_H231delinsRY enhanced the binding with PLRG1 (Figure 2E and Supplemental Figure 2D). For the PLRG1 colocalization analysis, the M230_H231delinsRY variant did not exhibit any notable differences from the WT, and no differences were observed for the other variants except for F412Lfs*17 (Supplemental Figure 2, E–G). The F412Lfs*17 mutant localized exclusively to the cytoplasm, while PLRG1 remained nuclear, suggesting PLRG1 localization is SNW1 independent and not sequentially recruited by SNW1.

For D205A, we observed multiple interactions between residue ASP205 and residues of PRPF8 within the spliceosome complex, including hydrophobic interactions with PHE481 and a salt bridge interaction with HIS121 (Figure 2F). As analyzed, D205A markedly diminished the binding between SNW1 and PRPF8 (Figure 2G and Supplemental Figure 2H). In summary, all assessed SNW1 patient mutations either affected protein expression or localization, or they influenced interactions with other proteins within the spliceosome complex. A summary of SNW1 variant types and their experimentally validated effects on splicing, protein expression, and interactions is provided in Supplemental Table 4. These results add value to the notion that these loss-of-function mutations affect the function of the splicing complex and further lead to neurodevelopmental damage.

### Assessing the role of SNW1 in brain development using Drosophila.

**Figure 3:**
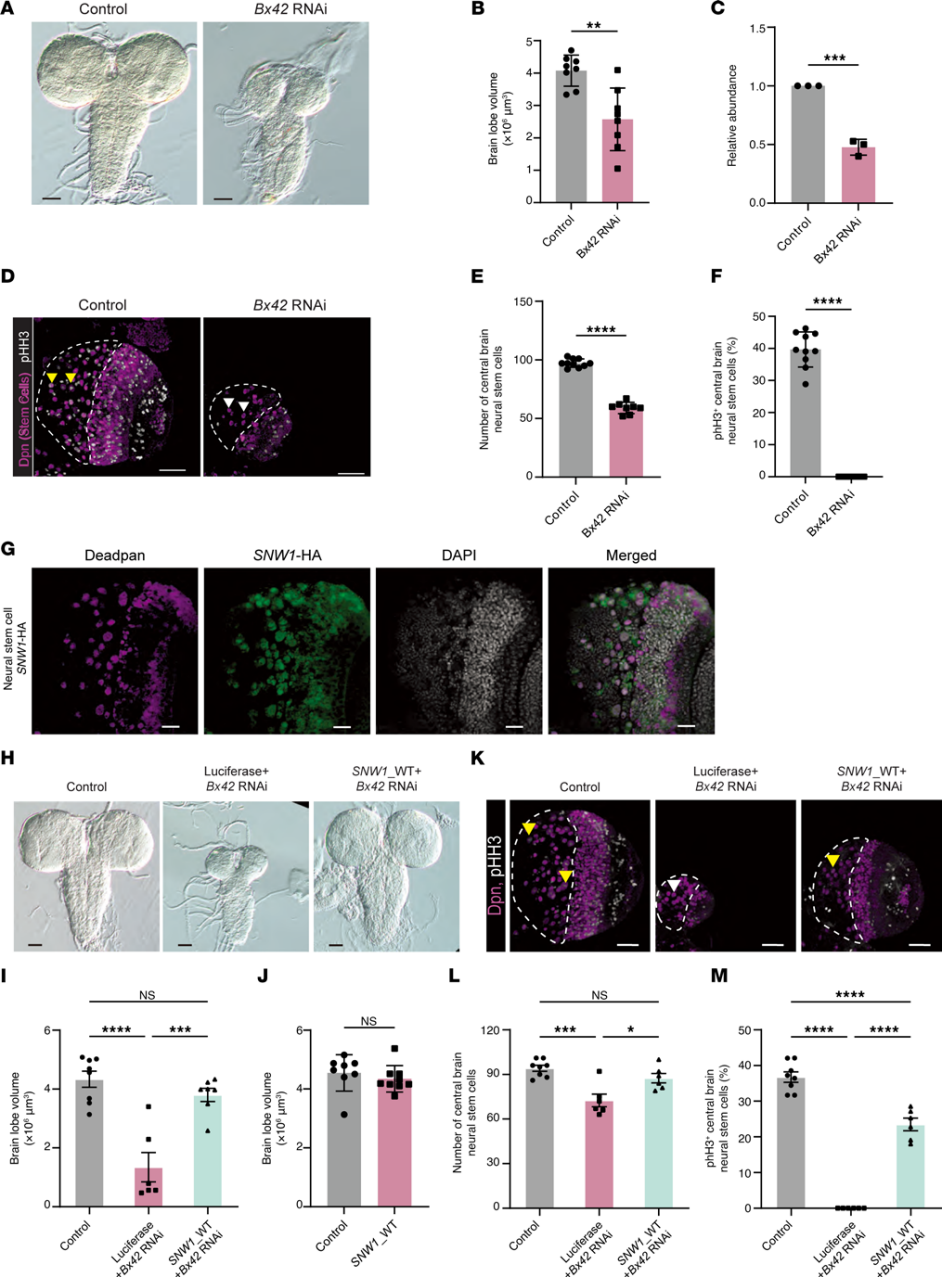
*Bx42* knockdown in NSCs leads to reduced brain lobe volume, stem cell number, and percent proliferating stem cells. (**A**) Images of third-instar larval brains with knockdown of *EGFP* or *Bx42* in NSCs (*insc-GAL4*). (**B**) The brain lobe volume of *EGFP* (control) and *Bx42* knockdown in NSCs. (**C**) *Bx42* transcript expression (normalized to *RpL32*) in control and *Bx42* RNAi third-instar brains. (**D**) Confocal images of a single brain lobe from third-instar larvae with knockdown of *EGFP* or *Bx42* in NSCs. Brains were stained for Deadpan (Dpn), a nuclear marker of NSCs, and p-HH3, a marker for proliferating cells. The central brain region is outlined in white. (**E**) The number of Dpn^+^ NSCs in the central brain region in *Drosophila* with *EGFP* (*n* = 10) and *Bx42* (*n* = 8) knocked down in NSCs. (**F**) Proliferating NSCs (yellow arrowheads in **D**) and non-proliferating (white arrowheads in **D**) in *EGFP*- (*n* =10) and *Bx42*-knockdown (*n* = 8) brains were quantified. Dpn^+^ cells without p-HH3 puncta are noted with white arrowheads in **D**. There was complete loss of proliferating NSCs in third-instar larvae with *Bx42* knockdown using *insc*-*GAL4*. (**G**) SNW1-HA was expressed in NSCs (*insc-GAL4*) and stained with Dpn (magenta), HA (green), or DAPI (white) to confirm presence of SNW1 protein in the nucleus of NSCs. (**H**) Images of third-instar larval brains with knockdown of *EGFP*, *Luciferase* + *Bx42* RNAi, or *SNW1*_WT *+ Bx42* RNAi in NSCs (*insc-GAL4*). (**I**) Brain lobe volume of genotypes from (**H**); each dot represents 1 brain. (**J**) The brain lobe volume of *Drosophila* expressing *SNW1* alone in NSCs was analyzed. (**K**) Confocal images of a single brain lobe from third-instar larvae with knockdown of *EGFP*, *Luciferase* + *Bx42* RNAi, or *SNW1*_WT + *Bx42* RNAi in NSCs. The central brain region is outlined in white. (**L**) Proliferating cells were quantified in *EGFP* (*n* = 8), *Luciferase* + *Bx42* RNAi (*n* = 6), and *SNW1*_WT + *Bx42* RNAi (*n* = 6) knockdown brains. (**M**) Proliferating NSCs (yellow arrowheads in **K**) and non-proliferating (white arrowheads in **K**) were quantified in *EGFP* (*n* = 8), *Luciferase* + *Bx42* RNAi (*n* = 6), and *SNW1*_WT + *Bx42* RNAi (*n* = 6) knockdown brains. Data are presented as mean ± SEM. For comparisons among 3 groups, ordinary 1-way ANOVA was performed followed by Tukey’s multiple-comparison test for post hoc pairwise analysis. For comparisons between 2 independent groups, 2-tailed unpaired Student’s *t* tests were used (without post hoc correction). *P < 0.05, **P < 0.01, ***P < 0.001, ****P < 0.0001. Scale bars: 50 μm (**A** and **H**), 40 μm (**D** and **K**), and 20 μm (**G**).

To investigate the in vivo function of SNW1, we used *Drosophila*, whose *SNW1* ortholog, *Bx42*, shares 60% amino acid identity, including the important SNW domain (42). To assess the necessity of *Bx42* in neural stem cells (NSCs), the GAL4-UAS system was used to knock down *Bx42* using RNAi. Male *EGFP* RNAi (control) or *Bx42* RNAi flies were crossed to female *inscuteable-GAL4* (*insc-GAL4*; a NSC driver) flies for cell-specific knockdown of *Bx42* in NSCs. Third-instar larval brains were dissected and stained with Deadpan (a NSC marker), and brain lobe volume was quantified. The knockdown of *Bx42* in NSCs resulted in a significantly reduced brain lobe volume compared with control (Figure 3, A and B; *P* = 0.0015). To ensure that the small brain lobe volume observed was due to the knockdown of *Bx42* transcripts, *Bx42* transcript levels were assessed using quantitative PCR (qPCR). Ubiquitous knockdown could not be validated since it is embryonic lethal. *Bx42* was knocked down using the *neuronal*-*driver*-*Synaptobrevin-GAL4* (*nSyb-GAL4*), as *Bx42* is normally expressed in neurons, and the abundance of neurons compared with neuroblasts makes the detection of knockdown more feasible. We found that the knockdown of *Bx42* resulted in a 55% reduction in the *Bx42* transcript, confirming that RNAi is functioning as expected (Figure 3C).

Using the same crosses and dissection methods described for brain lobe volume, third-instar larval brains were stained with Deadpan and phospho-histone H3 (p-HH3) to mark proliferating NSCs. The stereotyped number of NSCs in the central brain region of *Drosophila* larvae is around 100 cells, making it easy to quantify and observe changes in cell number (43–46). A significant reduction in the number of NSCs in the central brain region was found with *Bx42* knockdown compared with control (Figure 3, D and E; *P* < 0.0001). Additionally, the number of NSCs in the central brain region with p-HH3 puncta colocalized with Deadpan was counted. Interestingly, while 40% of NSCs in controls had positive p-HH3 puncta, there was a complete loss of proliferation in central brain NSCs with *Bx42* knockdown (Figure 3F; *P* < 0.0001).

To test conservation of protein function between *Drosophila* and humans, *SNW1* cDNA was expressed using the GAL4-UAS system to control expression. The *SNW1* construct is tagged with HA, so to confirm the presence of SNW1 protein, brains were stained for Deadpan and HA. SNW1-HA colocalized with Deadpan, indicating human SNW1 protein is stable in the NSC nucleus as expected (Figure 3G). Flies coexpressing *Luciferase* + *Bx42* RNAi (a GAL4 dilution control) had a significant reduction in brain lobe volume compared with control brains (Figure 3, H and I; *P* < 0.0001), similar to *Bx42* RNAi alone. These results indicate the addition of another UAS construct did not affect knockdown efficiency. Importantly, expression of *SNW1* + *Bx42* RNAi significantly rescued brain lobe volume compared with *Luciferase* + *Bx42* RNAi (Figure 3, H and I; *P* = 0.0002), indicating that human *SNW1* and *Drosophila* *Bx42* are functionally conserved. To verify that expression did not disrupt normal brain development, human *SNW1* was expressed in NSCs of WT *Drosophila*, and no differences in brain lobe volume were observed (Figure 3J; *P* = 0.4495), indicating no toxic effects. In addition, *Luciferase* + *Bx42* RNAi brains retained reduced NSC number (Figure 3, K and L; *P* = 0.0003) and a complete absence of proliferation (Figure 3, K and M; *P* < 0.0001), while coexpression of *SNW1* + *Bx42* RNAi rescued both stem cell number (Figure 3, K and L; *P* = 0.0121) and proliferation (Figure 3, K and M; *P* < 0.0001). Together, these results show that *Drosophila* *Bx42* and human *SNW1* are conserved and both influence brain development.

### Impact of SNW1 deletion on proliferation and apoptosis of NSCs in human brain organoids.

Since the loss of function of *SNW1* in *Drosophila* results in a microcephaly phenotype similar to that observed in patients, we utilized human embryonic stem cell–derived (hESC-derived) cerebral organoid (hCO) models to unveil the functions of *SNW1* in human brain development. Analysis of *SNW1* expression patterns in human and mouse fetal brains revealed a pronounced tissue-specific expression during neurodevelopment, with high levels observed in highly proliferating cells and minimal expression in mature neurons (Supplemental Figure 3, A and B). This suggests that SNW1 may play a functional role predominantly during the early stages of neurogenesis, rather than in the maintenance of mature neuronal function. Based on these findings, 2 heterozygous SNW1 knockout (*SNW1*^+/−^) hESC lines, designated nos. 1-2 and 2-4, were generated from H9 hESCs using CRISPR/Cas9 genome editing technique (Supplemental Figure 3C). Sanger sequencing and PCR electrophoresis confirmed successful heterozygous deletion of exons 4 and 5 in the *SNW1*^+/−^ hESC lines (Supplemental Figure 3, D and E). Subsequent Western blot analysis confirmed a significant reduction in *SNW1* expression in both 1-2 and 2-4 lines compared with H9 controls (Supplemental Figure 3F). To further investigate whether the deletion of *SNW1* caused any defects in hESCs, we performed immunostaining and alkaline phosphatase activity assays to check the pluripotency across the 3 cell lines. The results revealed no significant differences in these markers among the lines, indicating that the expression of core pluripotency markers remained unaffected (Supplemental Figure 3, G–I). The results suggest that *SNW1* deletion did not affect the differentiation potential of hESCs.

**Figure 4:**
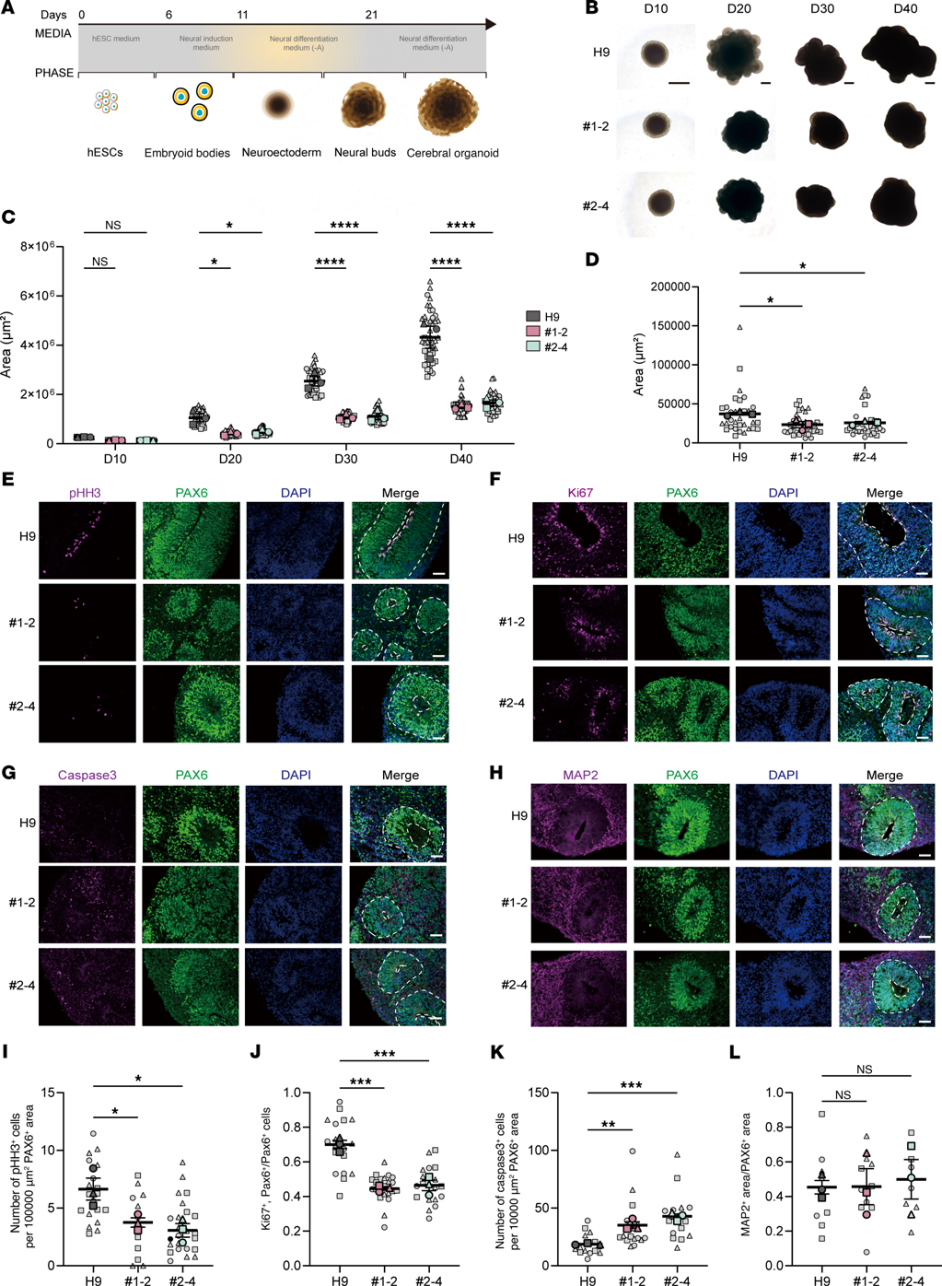
Developmental progression and phenotypic analysis of *SNW1*^+/–^ cerebral organoids reveals altered size and NSC properties. (**A**) Schematic of making cerebral organoids based on Lancaster methods. (**B**) Representation images of H9 and SNW1^+/–^ brain organoids cultured for 10, 20, 30, and 40 days. Scale bars: 500 μm. (**C**) The size of brain organoids from different groups was quantified at multiple time points across 3 independent experiments. (**D**) The ventricular zone–like (VZ-like) PAX6^+^ rosette area was quantified, with each data point representing an individual rosette. Data were collected from 3 independent experiments. (**E**–**H**) Immunofluorescent staining was performed on sections of WT and *SNW1*^+/–^ brain organoids cultured for 45 days. PAX6 was used to label the VZ-like rosette area, which was further stained with p-HH3 (**E**), Ki67 (**F**), Caspase3 (**G**), and MAP2 (**H**). The proliferating rosettes and apical surface adjusted to ventricle-like regions are highlighted by white dash lines. Scale bars: 50 μm. (**I**) Quantification of the ratio of p-HH3^+^ cells per 100,000 μm^2^ PAX6^+^ area of each rosette. Each plot represents an individual rosette. (**J**) Quantification of the ratio of Ki67 and PAX6 double-positive cells versus the total number of PAX6^+^ cells of each rosette. Each plot represents an individual rosette. (**K**) Quantification of the ratio of Caspase3^+^ cells per 100,000 μm^2^ PAX6^+^ area of each rosette. Each plot represents an individual rosette. (**L**) Quantification of the ratio of MAP2 and PAX6 double-positive cells versus the total number of PAX6^+^ cells of each rosette. Each plot represents an individual rosette. Small gray symbols represent size measurements of single hCOs (technical replicates), different symbol shapes denote 3 independent biological replicate experiments (*n* = 3 per group), and large colored symbols indicate means of technical replicates within each biological replicate. Data are presented as mean ± SEM. Statistical significance was tested by 1-way ANOVA. *P < 0.05, **P < 0.01, ***P < 0.001, ****P < 0.0001.

After validation of pluripotency by preservation of pluripotency markers, *SNW1*^+/−^ hESCs and H9 cells were further used to generate hCOs (Figure 4A). To determine if the alterations in proliferation observed in *Drosophila* models could be validated in humans, we measured the sizes of organoids at several time points within 40 days to trace the possible changes (Figure 4B). From day 20, hCOs derived from *SNW1*-KO lines exhibited a significant reduction in size compared with H9 controls, and this smaller volume phenotype persisted as the culture period progressed (Figure 4C). Following this observation, we further investigated the underlying mechanisms causing this developmental difference, focusing on the phenotypic characteristics of NSCs in 45-day-old hCOs, as observed in *Drosophila*. The rosette-like structures within hCOs, which closely resemble the early embryonic neural tube, are primarily composed of NSCs. We used PAX6 as a marker to delineate these structures and identify regions critical for NSC proliferation and differentiation. Quantitative analysis revealed a significant reduction in the area of PAX6^+^ regions in *SNW1*^+/−^ hCOs compared with controls, suggesting that partial loss of *SNW1* compromises the size of the NSC pool (Figure 4D). To assess the functional state of NSCs within these regions, we performed immunostaining for markers of proliferation (p-HH3 and Ki67), apoptosis (caspase 3), and neuronal differentiation (MAP2) (Figure 4, E–L). The proportion of cells coexpressing PAX6 and proliferation markers (p-HH3 or Ki67) was significantly decreased in *SNW1*^+/−^ organoids, indicating reduced proliferative capacity of NSCs (Figure 4, E, F, I, and J). Conversely, the frequency of caspase 3^+^ cells among the PAX6^+^ population was markedly increased, suggesting enhanced apoptosis in the absence of sufficient *SNW1* function (Figure 4, G and K). In contrast, the proportion of MAP2^+^ mature neurons showed no significant difference between *SNW1*^+/−^ and H9 control organoids, reinforcing the notion that SNW1 does not play a major role in the maintenance of differentiated neuronal populations (Figure 4, H and L). Together, these data collectively support a model in which SNW1 is critical for maintaining NSC homeostasis during early neurodevelopment, primarily by sustaining proliferation and inhibiting apoptosis, while not affecting the survival of mature neurons.

### SNW1 deficiency leads to widespread transcriptional alterations impacting neuronal differentiation and brain development.

**Figure 5:**
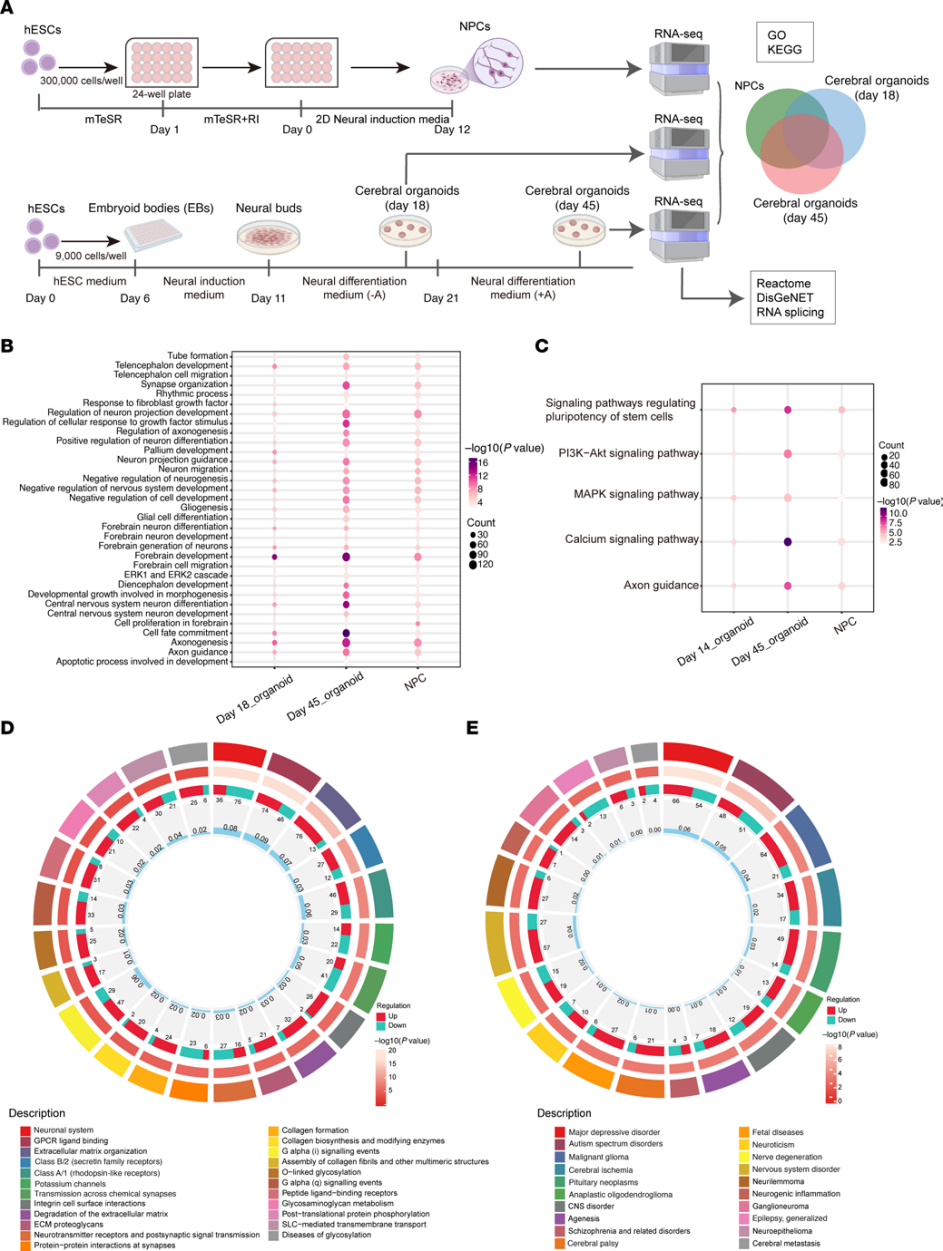
Developmental and transcriptomic analysis of human cerebral organoids derived from *SNW1*^+/–^ hESCs. (**A**) Schematic overview of the experimental design. hESCs were differentiated into NPCs over 12 days and into cerebral organoids over 45 days. NPCs cultured for 18 days, cerebral organoids cultured for 18 days, and cerebral organoids cultured for 45 days were collected for bulk RNA-seq analysis. (**B**) GO biological process analysis of DEGs is shown, highlighting common enriched terms across the 3 groups (NPCs, day 18 organoids, and day 45 organoids). (**C**) KEGG pathway enrichment analysis of DEGs is presented, displaying shared enriched pathways among the 3 groups (NPCs, day 18 organoids, and day 45 organoids). (**D**) Reactome pathway enrichment circos plot for 45-day cerebral organoids. For each Reactome term around the circle (outermost ring), the adjacent ring shows upregulated (red) versus downregulated (teal) gene counts; the next inner ring shows –log_10_(*P* value) as a heat map; and the innermost ring displays gene ratio bars. (**E**) DisGeNET disease association circos plot for 45-day cerebral organoids, plotted in the same concentric-ring format as in **D**.

SNW1 is both a transcriptional coregulator and a splicing factor, so its deficiency is highly likely to impact the expression of downstream target genes [192, 273]. To obtain a comprehensive understanding of how *SNW1* haploinsufficiency affects neural progenitor cells (NPCs), we performed bulk RNA-Seq analyses on NPCs differentiated for 12 days and cerebral organoids cultured for 18 and 45 days, derived from both WT and *SNW1*^+/–^ (no. 1-2) hESCs (Figure 5A). Individual analyses for each group are presented in Supplemental Figures 4–6. Gene Ontology (GO) and KEGG (Kyoto Encyclopedia of Genes and Genomes) pathway analyses revealed several commonly enriched biological processes and signaling pathways across all 3 developmental stages, including axonogenesis, forebrain development, the PI3K/Akt signaling pathway, and axon guidance (Figure 5, B and C). Notably, these enrichments were most prominent in 45-day cerebral organoids. Further pathway enrichment analysis using Reactome demonstrated that differentially expressed genes (DEGs) at this stage were predominantly associated with the neuronal system (Figure 5D). DisGeNET disease association analysis also revealed notable correlations with neuropsychiatric disorders, further underscoring the pivotal role of SNW1 in neurodevelopment (Figure 5E). Based on these findings, we focused our subsequent analyses on gene expression changes in the 45-day cerebral organoids.

In the 45-day cerebral organoids, we detected 3,572 DEGs (|log_2_FC| > 1 and *P*_adj_ < 0.05), of which 61.99% were upregulated (*n* = 2,214) and 38.01% were downregulated (*n* = 1,358) (Supplemental Figure 4C). Subsequent functional enrichment analyses revealed that these DEGs are substantially involved in several critical biological processes and signaling pathways, including axonogenesis, forebrain development, apoptotic processes, the neuroactive ligand-receptor interaction pathway, the PI3K/Akt signaling pathway, axon guidance, and others (Supplemental Figure 4, D and E). Moreover, genes involved in head development, embryonic morphogenesis, and neurological disorders were also markedly affected (Supplemental Figure 4, F and G). These results indicated that *SNW1* plays essential roles in neurodevelopment processes, which could be mapped to hCO development and phenotype manifestations in *Drosophila* models and patients, especially brain function and neurodevelopment.

### Disrupted AS integrity in SNW1^+/–^ human cerebral organoids.

To investigate the potential role of *SNW1* in NDDs associated with primary microcephaly, which is primarily caused by dysfunction in NPCs, we performed transcriptomic analysis and cell cycle assays on NPCs (Supplemental Figure 6). The results demonstrated that haploinsufficiency of *SNW1* (*SNW1*^+/−^) does not affect the NPC cycle (Supplemental Figure 6, G–I). Transcriptomic profiling revealed that *SNW1* haploinsufficiency predominantly disrupts RNA splicing processes and alters several signal transduction pathways (Supplemental Figure 6D). However, no enrichment was observed for transcription-related GO terms. Given that SNW1 and PPIL1 exhibit overlapping functions in RNA splicing and display phenotypic similarities, our investigation focused more on the posttranscriptional regulatory roles of SNW1, particularly its involvement in pre-mRNA splicing regulation.

**Figure 6:**
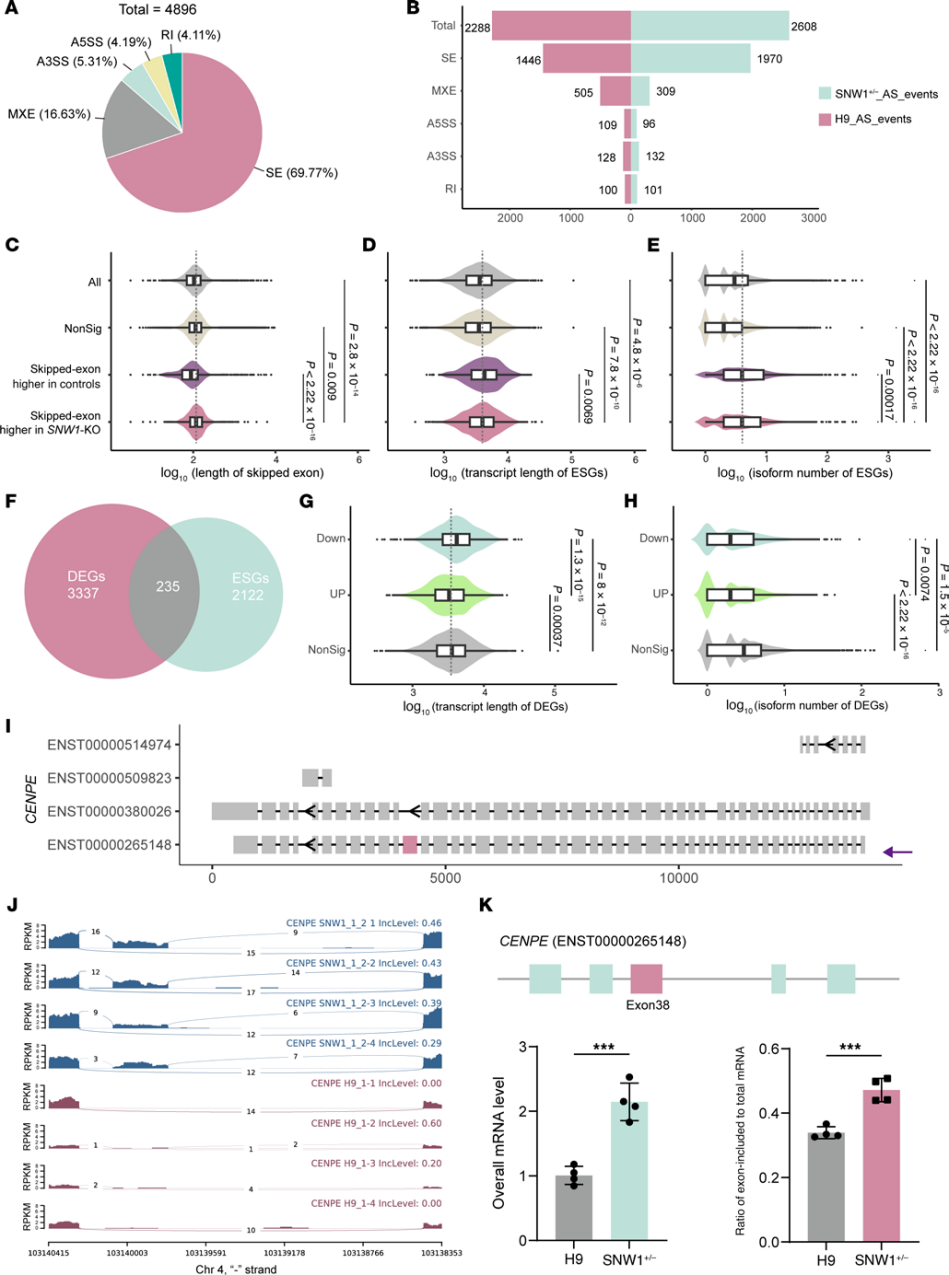
Splicing integrity defects in *SNW1*^+/–^ hCOs. (**A**) Impact of SNW1 depletion on 5 major types of AS events detected with rMATS in hCOs (45 days old). SEs were most affected, followed by mutually exclusive exons (MXE), alternative 5′ splice site (A5SS), alternative 3′ splice site (A3SS), and RI. (**B**) Columns showing numbers of significant events with higher inclusion level in *SNW1*^+/–^ (sky blue) or control (pink). (**C**) Distribution of differential splicing identified by rMATS in *SNW1*^+/–^ or control on the basis of SE length. Exons with long length show significantly higher SEs in *SNW1*^+/–^ hCOs. (**D**) Violin plot of ESGs with significant transcript lengths. (**E**) Violin plot of isoform numbers of ESGs. (**F**) Venn diagram of overlapping DEGs and ESGs. (**G** and **H**) Violin plot of transcript lengths of DEGs compared with nonsignificant genes. NonSig, nonsignificant genes (gray); UP, upregulated (light green); Down, downregulated (sky blue). (**I**) Schematic representation of human *CENPE* transcript isoforms. The purple arrow indicates the Matched Annotation from NCBI and EMBL-EBI (MANE)–selected canonical transcript. The identified exon in the rMATS analysis is marked in pink. (**J**) Sashimi plots of read density of *CENPE* transcript in 4 *SNW1*^+/–^ (no. 1-2) and 4 control brain organoids revealed that *SNW1*^+/–^ hCOs exhibited retention of exon 38, while the WT tended to skip this exon. (**K**) RT-qPCR validations of SE events of *CENPE* in WT and *SNW1*^+/–^ brain organoids (no. 1-2). Top: Schematic diagrams of *CENPE* transcript. The pink box represents the SEs. Bottom: Validation of significant SE events by semiquantitative RT-qPCR using *GAPDH* as reference gene. Relative level of overall mRNA (sky blue); ratio of exon-included mRNA to total mRNA (pink). Statistical significance was determined by Wilcoxon’s test. ***P < 0.001.

Comparative splicing analysis was performed using the rMATS pipeline between the 2 groups, revealing 4,896 significant AS events. Notably, skipped exon (SE) events represented the majority, accounting for 69.77% of the total (Figure 6A and Supplemental Figure 7, A and B). The markedly high frequency of SEs (1,970/1,446) observed in *SNW1*-KO hCOs stands out compared with other observed AS event ratios, while no significant difference was detected in retained intron (RI) events (Figure 6B). Compared with the control group, SE events in *SNW1*-KO hCOs exhibited a significant increase in the length of SEs, indicating that SNW1 deficiency induces aberrant splicing at longer exons (Figure 6C). There was no discernible difference in the preference for 5′ and 3′ splice sites among SE events (Supplemental Figure 7, C and D). SE and RI events are mediated by distinct molecular mechanisms involving the EJC pathways. The detection of SE defects suggests a regulatory role of SNW1 in a specific subset of splicing events. Furthermore, the SE events were particularly enriched in genes with a longer isoform and a higher number of isoforms, which we refer to as exon skipping genes (ESGs) (Figure 6, D and E). AS is highly prevalent and strictly conserved in the brain, contributing to the specificity and diversity of neural circuits, thereby resulting in genes with longer and more numerous isoforms (17, 18, 47, 48). These characteristics of ESGs also imply that *SNW1* deficiency renders the brain more susceptible compared with other tissues. Profiling of ESGs identified axon development as the most significantly disrupted module, reinforcing the phenotypes observed in flies and patients (Supplemental Figure 7, E and F).

During the mRNA processing in the presence of *SNW1* deficiency, SE splicing may lead to alterations in isoform ratios or destabilization of mRNA, thereby impacting gene expression. Among the 3,572 DEGs, 235 exhibited exon skipping (Figure 6F). Likewise, downregulated DEGs in *SNW1*-KO hCOs were significantly enriched for long genes, with an average of fewer than 8 annotated isoforms (Figure 6, G and H). These findings suggest that SNW1 is crucial for the proper spliceosomal processing of long mRNAs that are highly expressed in the brain. To validate our bioinformatics analysis, inclusion patterns of SE events in selected DEGs were validated by reverse transcription qPCR (RT-qPCR) (Supplemental Figure 8). Due to their roles in NDDs, we selected 11 candidate genes from the 235 genes identified, which are associated with NDDs or microcephaly according to the DisGeNET database. The results demonstrated that the overall mRNA levels of these genes in *SNW1*-KO hCOs changed significantly, as expected. For the SE events, primers were designed to measure the ratio of exon-included mRNA to the total mRNA, showing that most exhibited exon skipping (Supplemental Figure 8, B–I). This exon loss may result from aberrant splicing or the selection of noncanonical transcript isoforms, potentially explaining some of the observed differences in gene expression.

Among these genes, we selected 3 representative ones, all of which have been confirmed to be associated with neurodevelopment: *CENPE*, *MEF2C*, and *NRXN2* (Figure 6, I–K, and Supplemental Figure 9). For *CENPE*, there are currently 2 validated transcripts, with SE occurring in exon 38 of the canonical transcript (Figure 6, I–K). Similarly, in *SNW1*^+/–^ brains, exon 8 of the canonical transcript of *MEF2C* was skipped, whereas the canonical transcript represents the longest isoform (Supplemental Figure 9, A–C). Additionally, there were instances such as *NRXN2*, where multiple SE events occurred (Supplemental Figure 9, D–F). Based on bioinformatics analysis and qPCR validation, this was also attributed to differences in transcript selection between the 2 groups. Collectively, these findings indicate that *SNW1* deficiency compromises splicing fidelity, leading to substantial changes in splicing patterns and expression levels of genes critical for brain and neural development.

## Discussion

In this study, we identified 9 *SNW1* variants in individuals with NDDs accompanied by primary microcephaly, suggesting *SNW1* as a potential candidate gene for NDDs. Mechanistically, a series of in vitro functional assays supported the pathogenicity of these variants by demonstrating loss of *SNW1* function. The observed dysfunction was found to impair RNA splicing fidelity, suppress NSC proliferation, and induce apoptosis, ultimately contributing to a significant reduction in brain volume. These findings underscore the critical role of spliceosome integrity in neurodevelopment and highlight the importance of accurate splicing in neural development.

SNW1, a conserved component of the NTC, has been rarely studied in the context of neurodevelopment process. Here, through rigorous patient recruitment and variant screening, we identified a spectrum of *SNW1* variants in NDD patients, including start codon disruptions, missense mutations, splice site variants, and frameshift mutations. Despite their heterogeneity, the affected individuals exhibited overlapping clinical phenotypes, suggesting a shared pathogenic mechanism, likely mediated by splicing dysfunction. Functional analyses encompassing transcript processing, protein interaction, and subcellular localization provided direct evidence for the pathogenicity of these *SNW1* variants. However, given the rarity of these mutations, we cannot fully exclude the possibility that some variants are merely rare variants observed in the population. Additionally, the limited sample size constrained the statistical power for robust genotype-phenotype correlation analyses. Therefore, further validation in larger and independent cohorts is essential to establish the pathogenic relevance of these *SNW1* mutations in microcephaly. Moreover, the presence of epilepsy and autism spectrum behaviors in affected individuals raises the possibility that *SNW1* variants may disrupt neuronal network formation and function, leading to abnormalities in electrophysiological activity and synaptic plasticity. These clinical features align with SNW1’s core role in the spliceosome, where aberrant RNA splicing can dysregulate genes essential for neuronal differentiation, migration, and synapse formation (49, 50). While this hypothesis is supported by previous literature, it remains to be directly tested and represents a key direction for future research.

Then, to explore potential biological mechanisms underlying *SNW1* deficiency in NDDs, we established *SNW1*-knockdown models in *Drosophila* and cerebral organoids. Knockdown of the *SNW1* ortholog *Bx42* in *Drosophila* impaired CNS development, marked by reduced brain lobe size and loss of NSC proliferation. Notably, these phenotypes were rescued by reintroduction of human *SNW1*, indicating conserved function. Due to the lack of patient-derived cells, our study utilized a heterozygous knockout model based on hESCs, which may overlook patient-specific genetic modifiers. In cerebral organoids, *SNW1* haploinsufficiency recapitulated the microcephaly phenotype, characterized by impaired neural progenitor proliferation, increased apoptosis, and disrupted splicing of key neurodevelopmental genes. These observations underscore the fundamental role of neurogenesis — comprising proliferation, migration, and differentiation of NSCs — in cortical development (49). Perturbations in progenitor cell proliferation or apoptosis during neurogenesis often lead to changes in brain size (51). Beyond organoid and *Drosophila* models, *SNW1* knockdown also leads to smaller head structures and less developed forebrains in *Xenopus laevis* embryos and zebrafish, indicating evolutionarily conserved roles in early brain formation (22, 52). However, it is important to recognize limitations in model fidelity. *Drosophila* lacks mammalian brain architecture and circuits involved in higher cognition, while organoids lack vascularization, immune microenvironments, and long-term maturation required to study processes like synaptic pruning or myelination. Future research using patient-derived induced pluripotent stem cells or nonhuman primate models, combined with genome editing technologies, will allow us to introduce precise *SNW1* variants in endogenous contexts and further elucidate disease mechanisms.

Transcriptomic analysis of *SNW1*^+/–^ hCOs revealed widespread RNA splicing dysregulation, with notable enrichment of aberrant splicing events in genes essential for neurodevelopment (53). Previous research has also shown that deletion of SNW1 leads to rapid downregulation of p21 and renders cells more susceptible to p53-mediated apoptosis (31, 54). Our results demonstrated AS defects were particularly evident in genes like *CENPE* and *MEF2C*. *CENPE* primarily participates in precise chromosome segregation, kinetochore microtubule attachment, and mitotic checkpoint control. Defects in *CENPE* can lead to abnormal division of NPCs during development, identified previously as pathogenic in primary microcephaly (55, 56). Meanwhile, *MEF2C* is crucial for cardiac muscle formation and neuronal development and function. Research has linked *MEF2C* deficiency syndrome to moderate to severe intellectual disability, epilepsy, and language impairment, which was validated in animal models (57). Transcriptomic profiling identified ESGs as significantly enriched in axonal development, consistent with previous reports (18, 58). While our study connected spliceosomal defects with NDD pathogenesis, further experiments are required to validate causal relationships. It remains to be determined whether observed phenotypes are driven by specific mis-spliced genes such as *MEF2C* or *CENPE*, or if compensatory mechanisms attenuate splicing defects in vivo. Notably, some variants (e.g., c.-2_1del.) are predicted to abrogate translation initiation entirely, yet patients carrying these mutations do not show significantly different phenotypes from those with other variants. This raises the possibility of residual truncated protein expression or compensatory upregulation of *SNW1* paralogs, which may buffer the splicing defect and explain the partial discordance between molecular anomalies and clinical severity. Future efforts should focus on spatiotemporal analysis of splicing disruptions in patient-derived neural precursor cells to map dynamic effects and understand functional consequences.

Global splicing analysis further revealed that SNW1 deficiency particularly enriched SE events in long transcripts and genes with multiple isoforms. The developing brain expresses longer genes and exhibits highly active AS, relying on a sophisticated splicing regulatory network to ensure precise expression of neuron-specific isoforms. The observed SE events can result in either truncated mRNAs that escape nonsense-mediated decay or isoform ratio imbalances that destabilize gene function. However, these findings are limited by current transcript annotation databases, which have low 5′ and 3′ end coverage, especially for long or low-abundance transcripts. To overcome these limitations, we plan to implement long-read sequencing and single-cell transcriptomics to delineate full-length transcript isoforms and splicing heterogeneity in neural progenitors. Targeted validation using rapid amplification of cDNA ends PCR (RACE-PCR) will also be employed to confirm key splicing events. These technologies will enhance the precision of differential splicing analysis and facilitate more accurate genotype–phenotype correlations.

Importantly, our findings place SNW1 within a broader context of spliceosomal components implicated in NDDs. For instance, *SF3B1* mutations cause skeletal abnormalities, while *SF3B4* mutations lead to Nager syndrome (59, 60). Similarly, *EFTUD2* mutations result in mandibulofacial dysostosis with microcephaly (11, 12), and mutations in *PPIL1*, *PRP17*, and *PRPF19* are linked to neurodegenerative pontocerebellar hypoplasia (2, 14, 15). Notably, SNW1 deficiency shares overlapping phenotypes with PPIL1 and PRPF19 mutant patients, suggesting a core role of the NTC. Transcriptomic data suggest that spliceosomal mutations often exhibit locus-specific effects on splice site recognition, altering mRNA isoform diversity and contributing to distinct clinical phenotypes (61). The electron microscopy structure of the human spliceosome reveals that *SNW1* is essential for spliceosome function, especially exhibiting betweenness centrality within the B^act^ and ILS complexes (30, 32, 62, 63). SNW1 is located in the core region of the spliceosome, functioning similarly to a thread woven through its components. Several *SNW1* mutations have been identified in the interaction regions between SNW1 and spliceosome components such as PPIL1, PLRG1, and PRPF8, highlighting the regulatory role of *SNW1* within the spliceosome. Three mutations presented in this study demonstrate a complete loss of interaction with PPIL1. Although yeast and human spliceosomes share a set of evolutionarily conserved core spliceosomal proteins, human spliceosomal proteins often contain additional unstructured regions. The most important difference between them is the presence of the RNA helicase Aquarius and peptidyl prolyl isomerases (30). These findings emphasize the critical role of SNW1-PPIL1 interactions in splicing regulation and disease pathology.

Additionally, haploinsufficiency of *SNW1* was found to alter multiple signaling pathways beyond splicing. Previous studies in *Xenopus* and zebrafish have suggested that SNW1 may function as a scaffold in a scaffold in β-catenin, T cell factor, and lymphoid enhancer factor transcription factors–mediated (TCF/LEF-mediated) transcription, thereby regulating early neurodevelopmental factors such as SOX3, SNAI2, and EN2 (22, 52). SNW1 has also been implicated in Notch, BMP, and Wnt signaling during early embryogenesis (64-67). Therefore, it is plausible that SNW1 dosage affects signal transduction directly or indirectly through splicing perturbations. Further mechanistic studies are necessary to disentangle these interrelated effects.

In conclusion, our study elucidates the critical role of SNW1 in spliceosome function and its potential impact on NDDs. By providing insights into the molecular mechanisms underlying *SNW1*-related NDDs, our research paves the way for potential therapeutic strategies targeting spliceosomal function.

## Methods

The detailed materials and methods are provided in the Supplemental Methods. The antibodies and primers used in this study are listed in Supplemental Table 5 and Supplemental Table 6, respectively.

### Sex as a biological variable.

Sex was not evaluated as a biological variable. Both male and female flies were used in this study.

### Drosophila stocks.

The *Drosophila* strains were obtained from the *Drosophila* Genomics Resource Center. The following *Drosophila* lines were used: *Bx42* (the *Drosophila* homolog of *SNW1*) RNAi (*P{TRiP.HMC00086}attP40*), *EGFP* RNAi (*P{VALIUM22-EGFP.shRNA.attP40*), *insc*-*GAL4* (*P{w[+mW.hs]=GawB}insc[Mz1407]*), *nSyb-GAL4* (*P{y[+t7.7] w[+mC]=nSyb-GAL4.P}attP2*), and *UAS-Luciferase* (*P{UAS-SNW1-HA}VK37*) (68, 69). All flies were maintained at 25°C and grown on Archon Scientific glucose formula food in medium, wide, plastic vials. RNAi crosses were set at 29°C and grown on Archon glucose formula food with bromophenol blue added. Brain volume measurements and proliferation assessments were performed on wandering third-instar larval brains. The wandering third-instar larval stage was identified morphologically by extruding spiracles and gut clearance (70, 71).

### Cell culture.

HEK293T, HEK293, and HeLa cells were obtained from Cell Bank, Chinese Academy of Sciences. These cells were cultured in high-glucose DMEM (Gibco; catalog C11995500BT) supplemented with 10% FBS (Gibco; catalog 10091148) and 1% penicillin-streptomycin (Beyotime; catalog C0222). Feeder-free cells were cultured in mTeSR medium (Stemcell Technologies) with Matrigel (Corning). Feeder-dependent H9 hESCs were cultured on CF1-c-irradiated mouse embryonic fibroblasts with hESC medium containing DMEM/F-12 (Gibco), 0.5% GlutaMAX (Gibco), 20% KnockOut serum replacement (Gibco), 1% nonessential amino acid solution (MEM-NEAA; Gibco), and 0.1 mM β-mercaptoethanol (Amresco), with freshly added 4 ng/mL bFGF (Sino Biological). Detailed procedures were previously described (72).

### Generation of hCOs.

The H9 hESC line used in this study was preserved from laboratory stocks. The *SNW1* knockout cell line was constructed in H9 hESCs using CRISPR/Cas9 gene editing technology. To delete *SNW1*, 2 guide RNAs targeting the introns flanking exons 4 and 5 were designed and cloned into plasmids, which were then transfected into H9 hESCs via the Neon transfection system (Invitrogen). Through homologous recombination, eGFP replaced exons 4 and 5 of SNW1, and GFP^+^ *SNW1*-KO cells (heterozygous) were identified by FACS.

hCOs were established following the protocol published by Lancaster (73). H9 hESC or *SNW1*-KO cells were treated with EDTA and Accutase (Invitrogen) to form single-cell solutions to generate embryoid bodies (EBs). Then, 9,000 cells were plated into each well of a low-binding 96-well plate in mTeSR medium with ROCK inhibitors (Selleck). After 3 days, fresh mTeSR without ROCK inhibitors was added. On day 5, EBs were transferred to neural induction media, which was refreshed every 2 days for 6 days. The neural induction media consisted of DMEM/F-12 (Gibco) supplemented with 1% N2 Supplement (Gibco), 1% l-GlutaMAX (Thermo Fisher Scientific), 1% MEM-NEAA (Gibco), and 0.01% heparin solution (Stemcell Technologies). Subsequently, EBs were embedded in Matrigel droplets and cultured in differentiation medium without vitamin A. The differentiation medium consisted of 50% DMEM/F-12 (1:1) and 50% Neurobasal medium (Gibco) supplemented with 1% N2 supplement, 1% B27 supplement minus vitamin A (Invitrogen), 1% GlutaMAX, 1% MEM-NEAA, 0.1 mM β-mercaptoethanol, 20 ng/mL EGF, and 20 ng/mL FGF-2. After 10 days, droplets were transferred to medium with vitamin A on an orbital shaker. Media were changed every 7 days, and the morphological appearance of organoids in both groups was examined.

### Statistics.

All power analyses were done with the G*Power program (https://www.psychologie.hhu.de/arbeitsgruppen/allgemeine-psychologie-und-arbeitspsychologie/gpower) post hoc to assess the power of our *n* sizes. Data are presented as mean ± SEM. Unless otherwise specified in figure legends, the following statistical tests were applied: For comparisons among 3 groups, ordinary 1-way ANOVA was performed followed by Tukey’s multiple-comparison test for post hoc pairwise analysis. For comparisons between 2 independent groups, 2-tailed unpaired Student’s *t* tests were used (without post hoc correction). Statistical significance was defined as *P* less than 0.05.

### Study approval.

The patients included in this study were from 7 unrelated families, originating from various regions around the world, including China, France, Germany, and the United States. Information regarding gender, age, and health status is listed in Supplemental Tables 1 and 2. All work involving patients was approved by the ethics committee of the Maternal and Child Health Hospital of Hunan Province (2024-S043) and conducted in accordance with established guidelines. Consent forms were signed by all patients or their guardians, explicitly permitting the use of the patients’ photographs in this study.

### Data availability.

The raw sequence data reported in this paper have been deposited in the Genome Sequence Archive of the National Genomics Data Center, China National Center for Bioinformation/Beijing Institute of Genomics, Chinese Academy of Sciences (GSA-Human: HRA012051 and HRA008110), publicly accessible at https://ngdc.cncb.ac.cn/gsa-human All underlying numerical data used to generate the graphs and statistical analyses in this study are provided in the Supporting Data Values file.

## Author contributions

The number of experiments performed by each researcher was the method used for assigning the order of the 3 co–first authors. LJ led the molecular experiments and manuscript writing, with Keyi Li, ZL, DR, KY, YL, and FY providing assistance. JY and JX conducted the organoid-related experiments, with support from ZG, GW, ZX, YW, HZ, and SB. NAL performed the *Drosophila* experiments, assisted by AB and NL. HW and LL were responsible for the bioinformatics analysis. HP, JH, JZ, RAJ, PZ, LM, FF, PC, CZ, NC, LR, AMCG, HSNF, ET, NMG, XL, WL, LAF, AYH, LZ, AM, ES, CSH. KK, WG, PW, and Kai Li contributed to providing and analyzing the clinical data of patients. The corresponding authors, SB, GH, and XM, supervised the project design and revised the manuscript.

## Supplementary Material

Supplemental data

Unedited blot and gel images

Supporting data values

## Figures and Tables

**Table 1:**
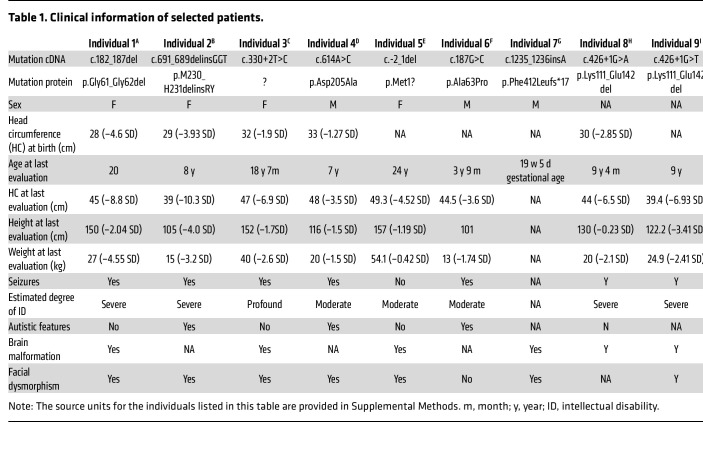
Clinical information of selected patients.

## Version 1 07/03/2025

In-Press Preview

## Version 2 09/16/2025

Electronic publication
