# Supplementary material for: Mutations in the spliceosomal gene SNW1 cause neurodevelopment disorders with microcephaly
Source: J Clin Invest. 2025 Jul 3;135(18):e186119. doi: 10.1172/JCI186119 (PMC12435841; doi:10.1172/JCI186119)
Supplement: Supplemental data [file jci-135-186119-s227.pdf]

Supplementary Figures 1-9

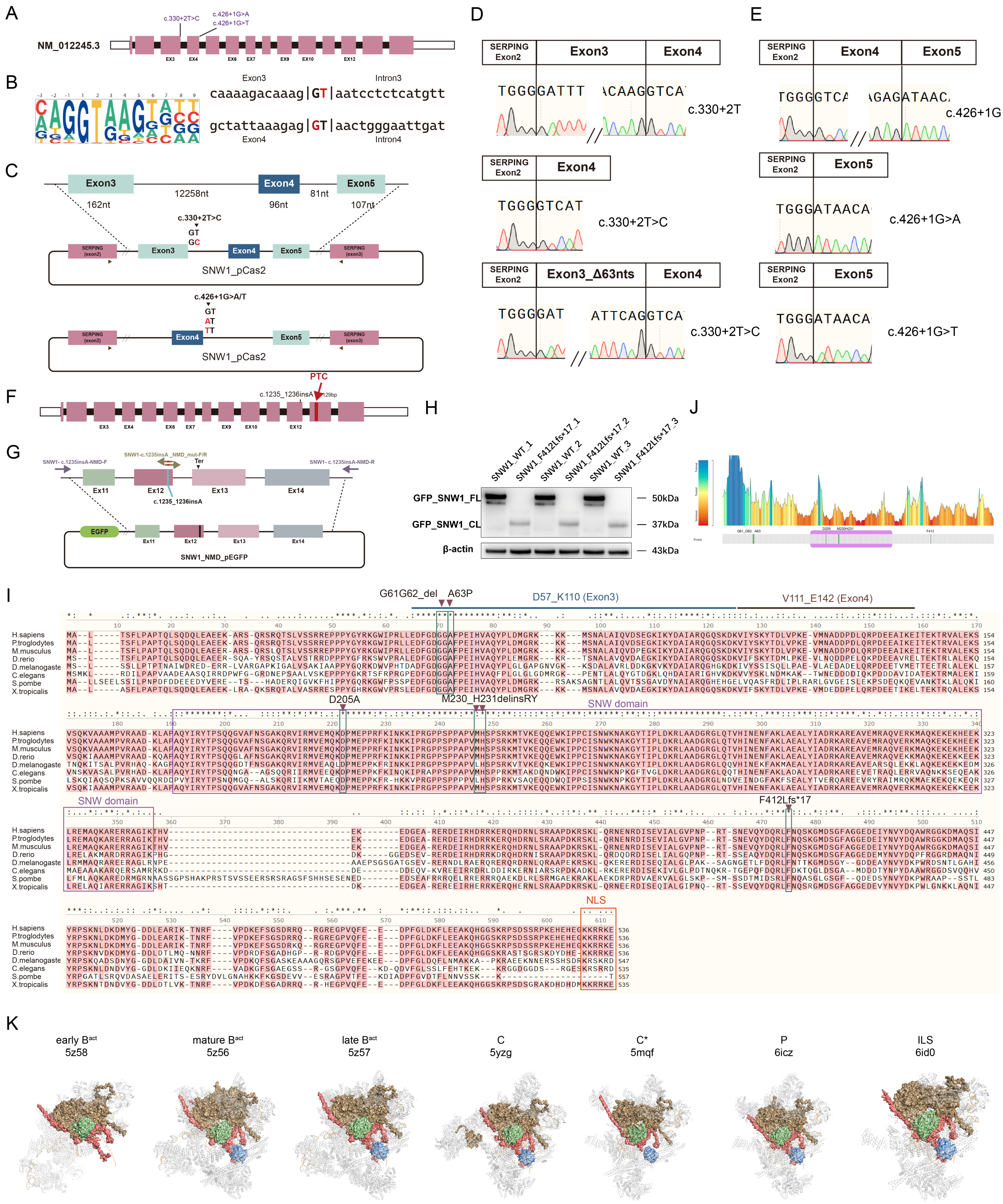

Supplementary Figure 1 Characterization of SNW1 variants and their impact on splicing, protein Function, and conservation.

(A) Diagram showing the locations of c.330+2T>C and c.426+1G>A/T variants on the *SNW1* transcript. (B) Comparison of the canonical splice donor site sequence with the donor site sequences of exon 3 and exon 4 of *SNW1*. (C) Structure of the

minigene *SNW1*_exons3-5 for *SNW1* c.330+2 T>C:and c.426+1G>A/T. cDNA products were amplified using primers situated in SERPING Exons 2 and 3, is represented with brown triangles. (D) The c.330+2T>C construct was validated by Sanger sequencing to exhibit complete skipping of exon 3 and partial 63 bp skip. (E) The variant constructs c.426+1G>A and c.426+1G>T were validated by Sanger sequencing to exhibit complete skipping of exon 4. (F) Schematic diagram of the variant c.1235_1236insA on the *SNW1* transcript. (G) Schematic diagram of *SNW1*_c.1235_1236insA NMD minigene reporter based on pEGFP-C3. (H) Western blotting for SNW1 in lysates obtained from HEK293T cells transfected with SNW1 WT or F421Lfs*17 mutated vectors. FL: full length; CL: cleaved length. (I) Alignment of SNW1 amino acid sequence, showing conservation across species: *Homo sapiens* (NP_036377.1), *Pan troglodytes* (XP_510097.2), *Mus musculus* (NP_079783.2), *Danio rerio* (NP_001002864.1), *Drosophila melanogaster* (NP_001259370.1), *Caenorhabditis elegans* (NP_505950.1), *Schizosaccharomyces pombe* (NP_588213.1), *Xenopus tropicalis* (NP_001017145.1). Conserved residues are highlighted in pink, where "*" indicates fully conserved residues, ":" indicates highly conserved residues, and "." indicates moderately conserved residues. The sequence alignment was performed using the T-Coffee tool (https://tcoffee.crg.eu/apps/tcoffee/index.html). The green boxes indicate the variant sites identified in this study, while the purple and orange regions correspond to the SNW domain and the nuclear localization signal, respectively. (J) An intolerance landscape plot generated by MetaDome (https://stuart.radboudumc.nl/metadome/dashboard) for *SNW1* variant (NM_012245.3) analysis. Exonic variants highlighted in green represent those identified in the current study. (K) Cryo-EM structures of human spliceosomal complexes showing the binding of SNW1 (surface in pink), PRPF8 (surface in brown), PLRG1 (surface in olive drab) to PPIL1 (surface in sky blue). The spliceosomal complexes shown include early B^{act} (PDB: 5Z58), mature B^{act} (PDB: 5Z56), late B^{act} (PDB: 5Z57), C (PDB: 5YZG), C* (PDB: 5MQF), P (PDB: 6ICZ), and ILS (PDB: 6ID0).

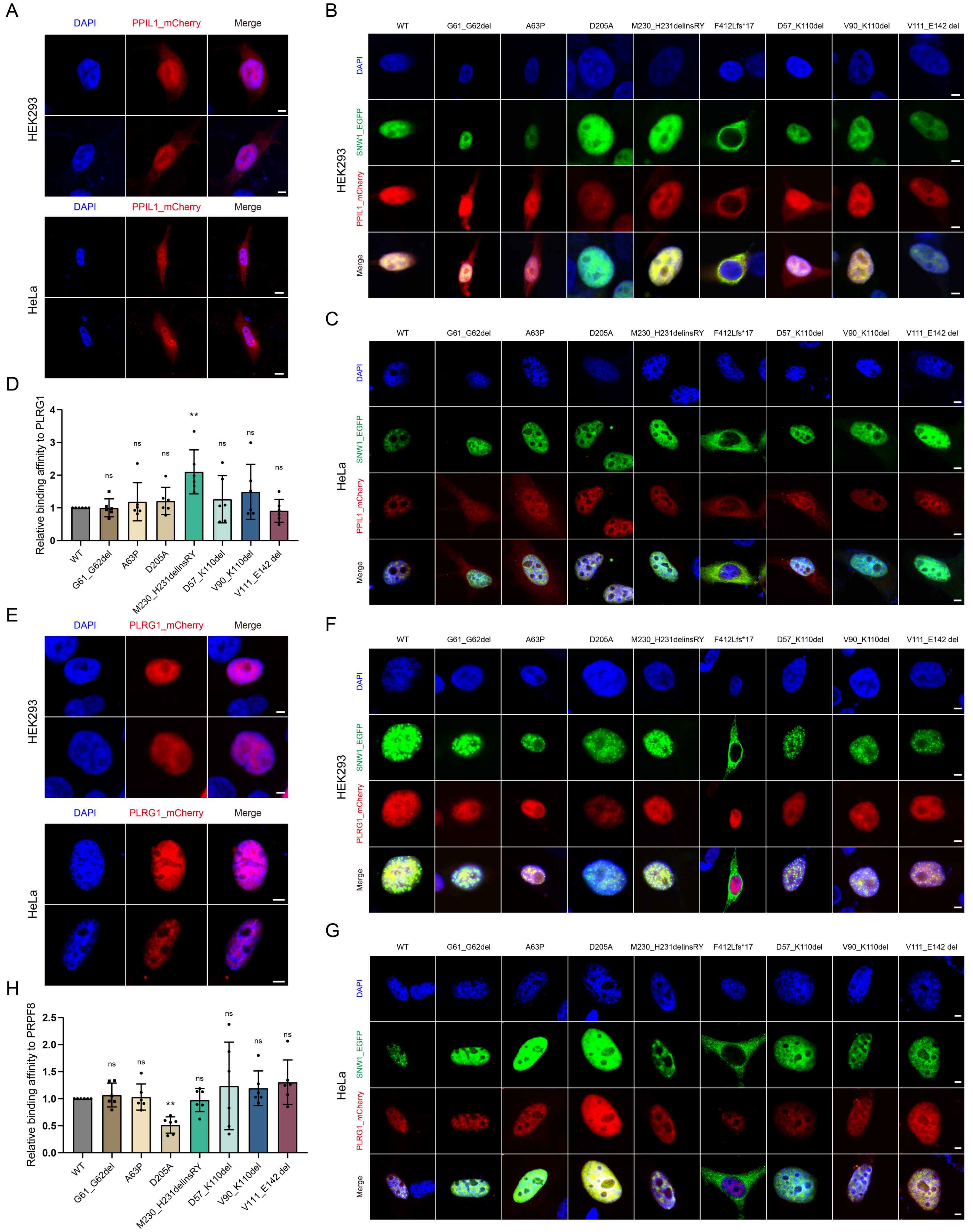

Supplementary Figure 2 Subcellular localization and interaction analysis of SNW1 variants with PPIL1, PLRG1, and PRPF8.

(A) Subcellular localization of mCherry-tagged PPIL1 in HEK293cells (top, scale bars = 2.5 μm) and HeLa cells (bottom, scale bars = 10 μm). Fluorescence images show PPIL1 (red) and DAPI (blue). (B-C) Effects of SNW1 mutations on the co-localization of SNW1 and PPIL1 in (B) HEK293 cells (scale bars = 2.5 μm), and (C) HeLa cells (scale bars = 5 μm).

Fluorescence images were captured using laser scanning confocal microscopy (Leica TCS SP8) with 63 × oil glass. SNW1 (green), PPIL1 (red), and DAPI (blue) are displayed. (D) Quantitative analysis of the binding affinity between SNW1 variants and PLRG1. Data are presented as mean ± SEM from three independent experiments (IP-Flag/IP-HA), with a total of six data points. (E) Subcellular localization of mCherry-tagged PLRG1 in HEK293cells (top, scale bars = 2.5 μm) and HeLa cells (bottom, scale bars = 5 μm). Fluorescence images show PLRG1 (red) and DAPI (blue). (F-G) Effects of SNW1 mutations on the co-localization of SNW1 and PLRG1 in (F) HEK293 cells (scale bars = 2.5 μm), and (G) HeLa cells (scale bars = 5 μm). Fluorescence images were captured using laser scanning confocal microscopy (Leica TCS SP8) with 63 × oil glass. SNW1 (green), PLRG1 (red), and DAPI (blue) are displayed. (H) Quantitative analysis of the binding affinity between SNW1 variants and PRPF8. Data are presented as mean ± SEM from three independent experiments (IP-Flag/IP-HA), with a total of six data points. ** $P < 0.01$, ns, no significance.

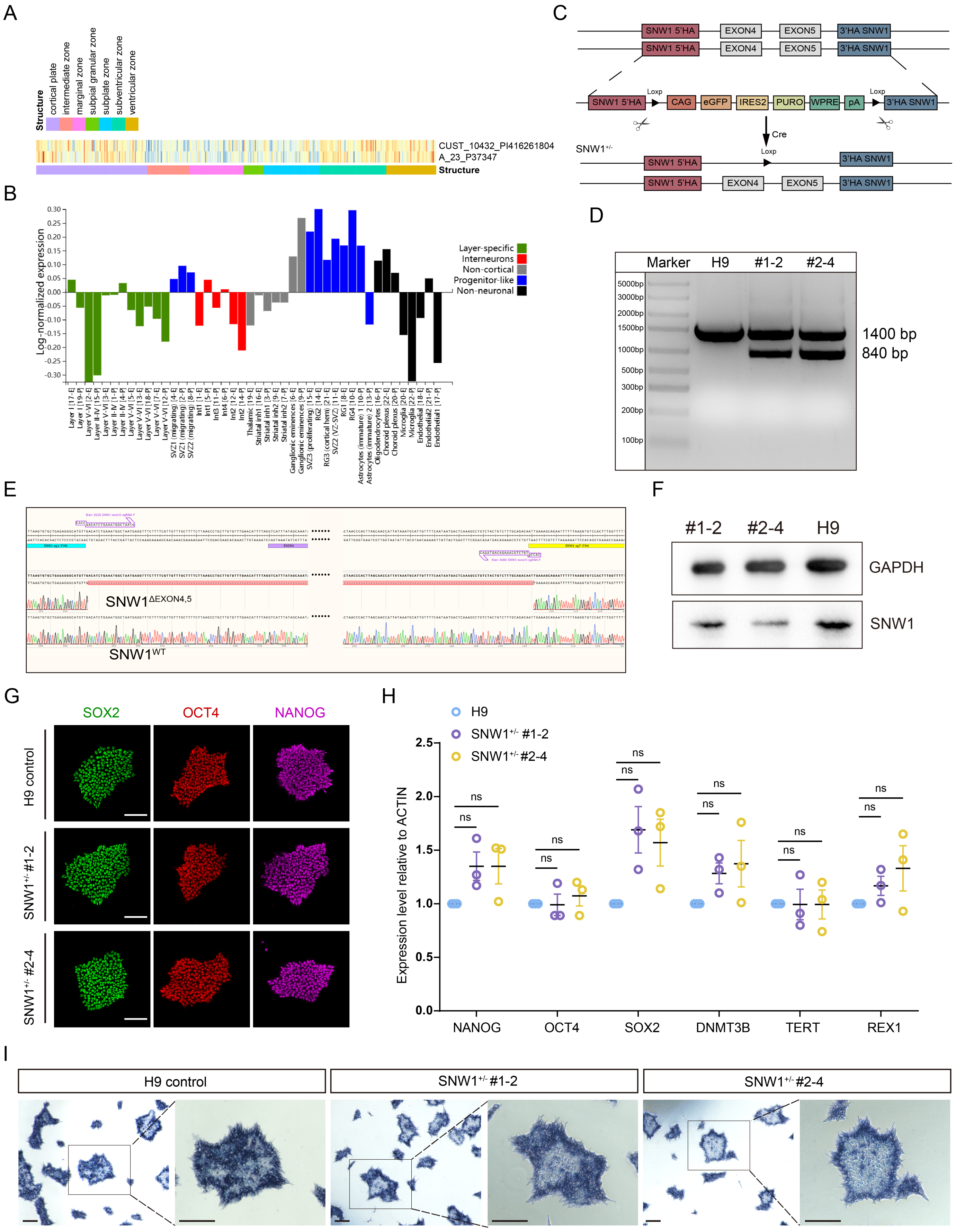

Supplementary Figure 3 Expression, generation, and characterization of *SNW1*$^{+/-}$ human embryonic stem cells (hESCs).

(A) Expression pattern of *SNW1* in the developing human fetal cortex at 21 post-conception weeks (pcw) (https://human.brain-map.org/). (B) Single-cell RNA-seq data of the mouse cortex at E14.5, showing specific expression of *SNW1* in neural

progenitor cells (https://zylkalab.org/datamousecortex). (C) Schematic of the generation of SNW1$^{+/-}$ hESC line. Step-by-step procedure can be found in the Materials and Methods section. (D) Schematic diagram of PCR electrophoresis results for genotyping. wildtype: 1400 bp, *SNW1*$^{+/-}$: 840 bp. (E) Schematic of sanger sequencing results of wildtype alle and *SNW1*$^{+/-}$ in *SNW1* locus. (F) The Western blots result showed the downregulation of SNW1 protein in *SNW1*$^{+/-}$ hESC lines. Proteins were collected from wildtype H9, *SNW1*$^{+/-}$ (#1-2 and #2-4) hESCs. GAPDH were used as references. (G) Immunofluorescent staining was used to detect pluripotent gene expression in H9, *SNW1*$^{+/-}$ (#1-2 and #2-4) hESCs. Embryonic stem cell markers SOX2, OCT4 and NANOG were expressed positively. Scale bars = 100 µm. (H) qPCR analysis of the expression of stem cell pluripotency markers on H9, *SNW1*$^{+/-}$ (#1-2 and #2-4) hESCs lines. No significant differences were observed in the expression levels of these markers among the cell lines Expression levels were measured relative to ACTIN of H9 control group. Each dot represented RNA collected from an independent batch of cells (n=3). (I) Alkaline phosphatase activity staining results of H9, *SNW1*$^{+/-}$ (#1-2 and #2-4) hESCs. Alkaline phosphatase activity, a key indicator of pluripotency in embryonic stem cells, was strongly positive in all cell lines, further confirming their undisturbed pluripotent state Scale bars = 200 µm. ns, no significance.

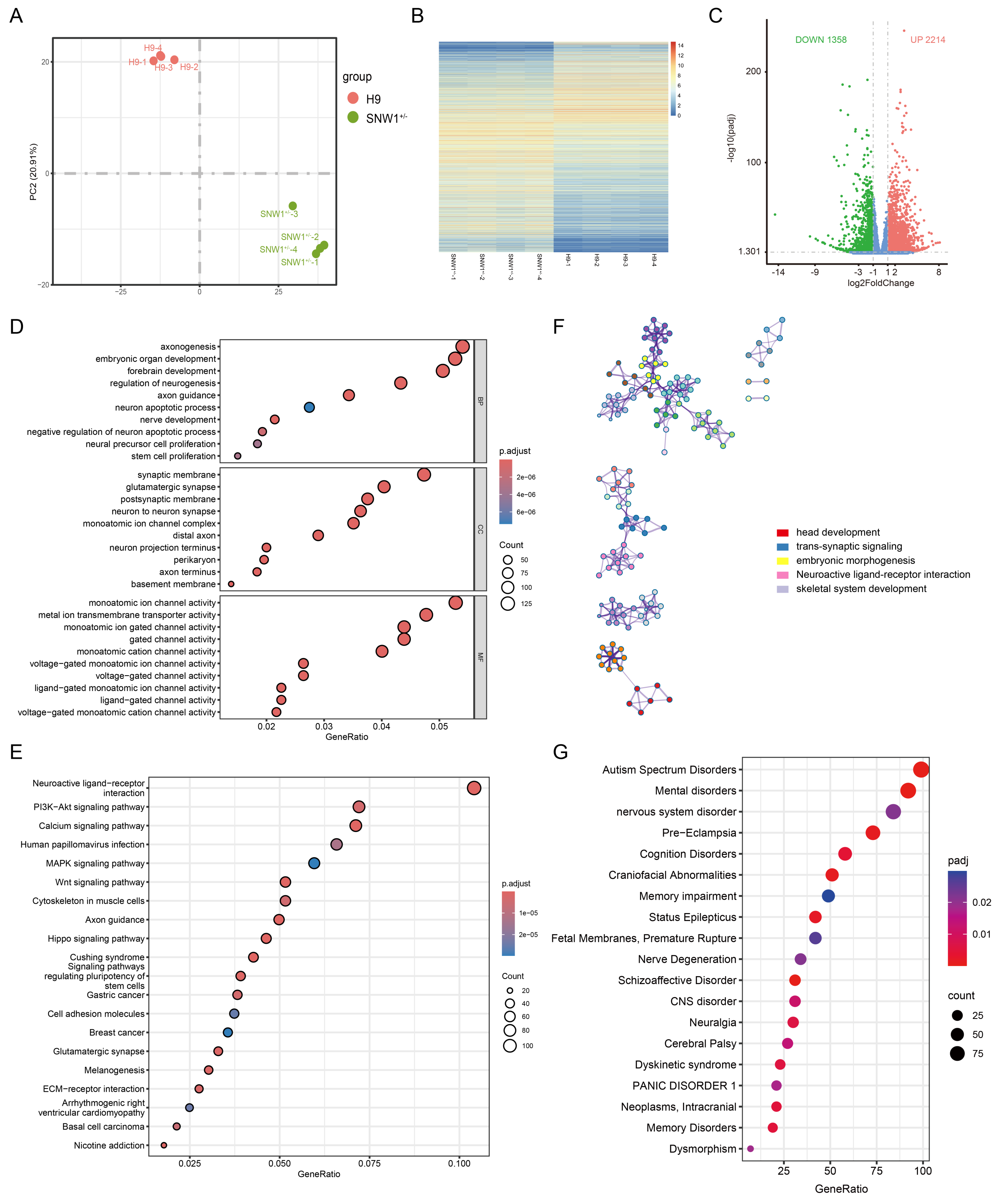

Supplementary Figure 4 Transcriptomic analysis of *SNW1*$^{+/-}$ cerebral organoids at day 45.

(A) Principal component analysis of differentially expressed genes between 45-day-old wildtype and *SNW1*$^{+/-}$ (#1-2) brain organoid based on RNA-seq. Each plot represented an individual organoid sample (sample n=3 for each group from 1 experiment, 3 organoids were pooled into one sample). (B) Heatmap representing hierarchical cluster of 3572 DEGs (|log2FoldChange| > 1, padj < 0.05) between wildtype and *SNW1*$^{+/-}$ (#1-2) brain organoids. Principal component analysis and clustering analysis confirmed intra-group consistency and reproducibility. (C) A volcano plot illustrating downregulated (blue) and upregulated (red) DEGs between wildtype and *SNW1*$^{+/-}$ brain organoids (#1-2). (D-G) GO term (D), network (F), KEGG

(E), and DisGeNET enrichment analysis of DEGs between wildtype and *SNW1*$^{+/-}$ (#1-2) brain organoids.

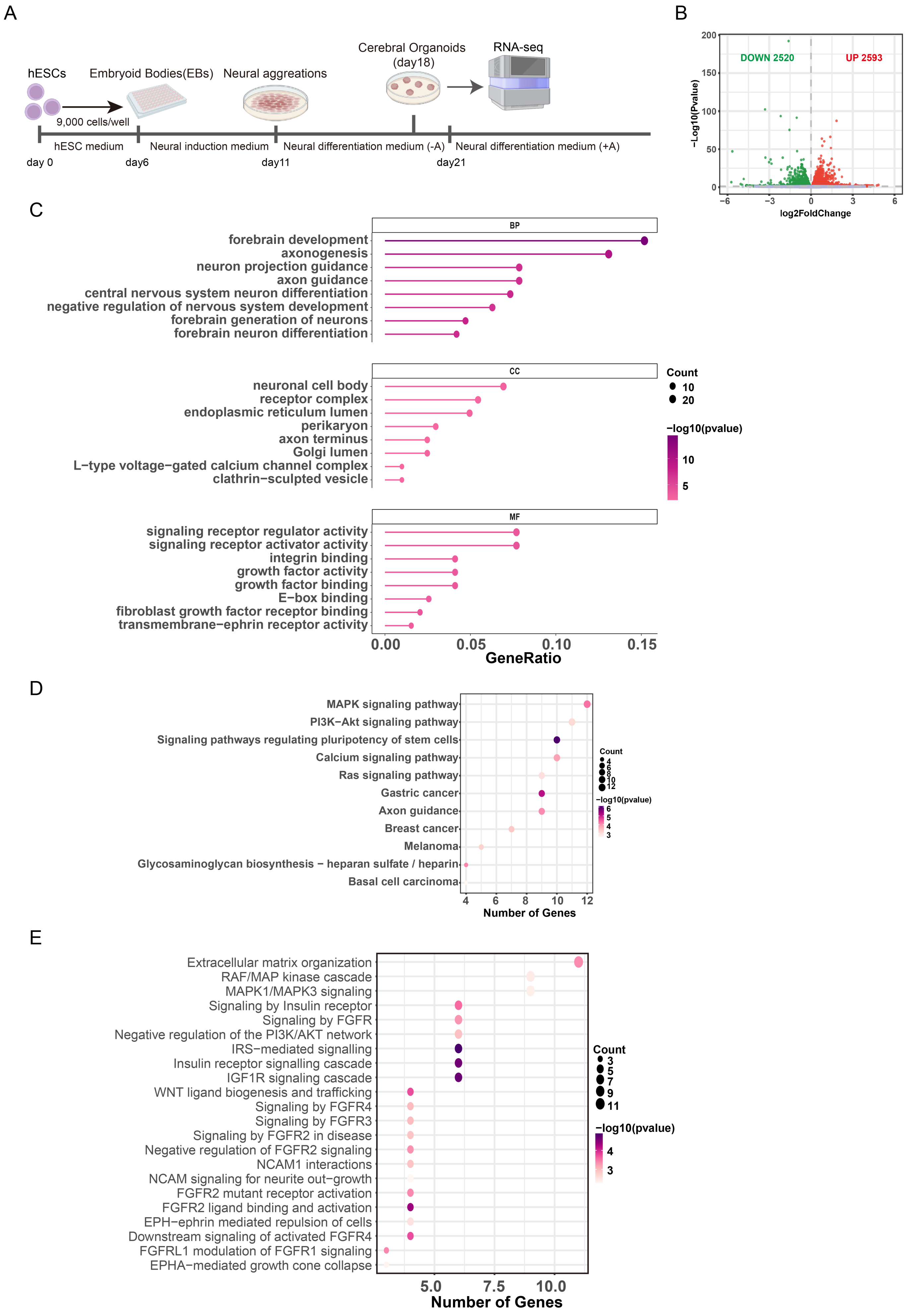
A
hESCs
Embryoid Bodies(EBs)
Neural aggreations
Cerebral Organoids (day18)
RNA-seq
9,000 cells/well
hESC medium
Neural induction medium
Neural differentiation medium (-A)
Neural differentiation medium (+A)
day 0
day6
day11
day21
B
DOWN 2520
UP 2593
−Log10(Pvalue)
log2FoldChange
C
BP
forebrain development
axonogenesis
neuron projection guidance
axon guidance
central nervous system neuron differentiation
negative regulation of nervous system development
forebrain generation of neurons
forebrain neuron differentiation
CC
neuronal cell body
receptor complex
endoplasmic reticulum lumen
perikaryon
axon terminus
Golgi lumen
L−type voltage−gated calcium channel complex
clathrin−sculpted vesicle
MF
signaling receptor regulator activity
signaling receptor activator activity
integrin binding
growth factor activity
growth factor binding
E−box binding
fibroblast growth factor receptor binding
transmembrane−ephrin receptor activity
Count
10
20
−log10(pvalue)
GeneRatio
D
MAPK signaling pathway
PI3K−Akt signaling pathway
Signaling pathways regulating pluripotency of stem cells
Calcium signaling pathway
Ras signaling pathway
Gastric cancer
Axon guidance
Breast cancer
Melanoma
Glycosaminoglycan biosynthesis − heparan sulfate / heparin
Basal cell carcinoma
Count
−log10(pvalue)
Number of Genes
E
Extracellular matrix organization
RAF/MAP kinase cascade
MAPK1/MAPK3 signaling
Signaling by Insulin receptor
Signaling by FGFR
Negative regulation of the PI3K/AKT network
IRS−mediated signalling
Insulin receptor signalling cascade
IGF1R signaling cascade
WNT ligand biogenesis and trafficking
Signaling by FGFR4
Signaling by FGFR3
Signaling by FGFR2 in disease
Negative regulation of FGFR2 signaling
NCAM1 interactions
NCAM signaling for neurite out−growth
FGFR2 mutant receptor activation
FGFR2 ligand binding and activation
EPH−ephrin mediated repulsion of cells
Downstream signaling of activated FGFR4
FGFRL1 modulation of FGFR1 signaling
EPHA−mediated growth cone collapse
Count
−log10(pvalue)
Number of Genes

Supplementary Figure 5 Transcriptomic analysis of *SNW1*$^{+/-}$ cerebral organoids at day 18.

(A) hESCs were cultured into cerebral organoids for 18 days, followed by bulk RNA-seq performed on both the H9 and *SNW1*$^{+/-}$ (#1-2) groups. (B) Volcano plot of differentially expressed genes. (C) GO term enrichment dot plot of differentially expressed genes. (D) The KEGG pathway enrichment dot plot of differentially expressed genes. (E) Reactome enrichment dot plot of differentially expressed genes.

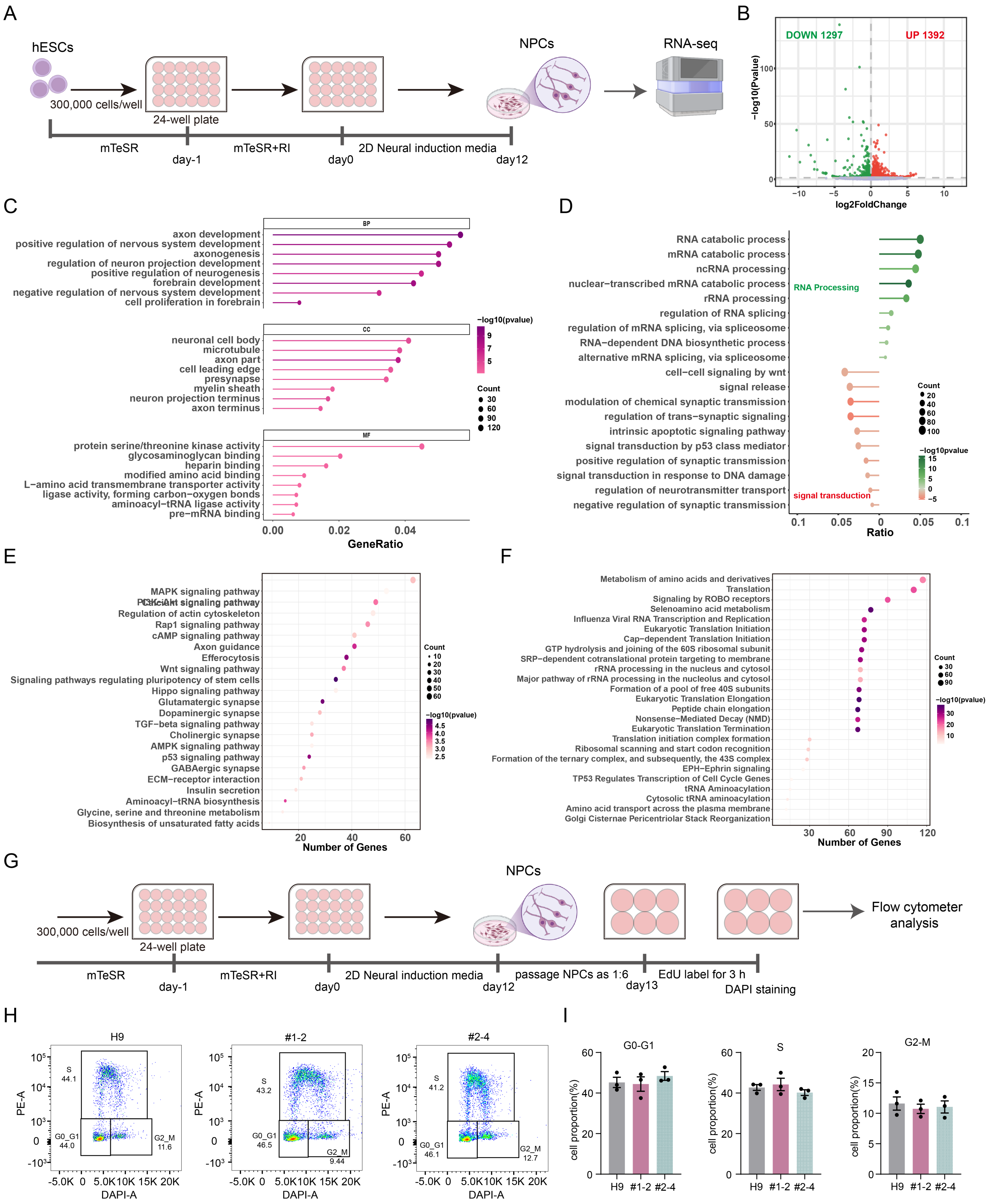

Supplementary Figure 6 Transcriptomic profiling and cell cycle analysis of *SNW1*$^{+/-}$ neural progenitor cells (NPCs).

(A) hESCs were differentiated into NPCs over 12 days, followed by bulk RNA-seq performed on both the H9 and *SNW1*$^{+/-}$ (#1-2) groups. (B) Volcano plot of differentially expressed genes between wildtype H9 and *SNW1*$^{+/-}$ (#1-2) NPCs. (C) The GO term enrichment dot plot based on differentially expressed genes between wildtype H9 and *SNW1*$^{+/-}$ (#1-2) NPCs. (D) GO Biological Process enrichment highlighting two major categories: RNA processing (green) and signal transduction (salmon). Bars represent gene ratio (DE genes/total annotated genes), bubble size is gene count, and color shows -log10 *P*-

value. (E) The KEGG pathway enrichment dot plot based on differentially expressed genes between wildtype H9 and *SNW1*$^{+/-}$ (#1-2) NPCs. (F) Reactome enrichment dot plot based on differentially expressed genes between wildtype H9 and *SNW1*$^{+/-}$ (#1-2) NPCs. (G) Schematic overview of cell cycle detection. Human embryonic stem cells (hESCs) were differentiated into neural progenitor cells (NPCs) over 12 days. After passage, NPCs were labeled by EdU for 3 hours, then stained by DAPI before analyzing by flow cytometer. (H) The distribution and cellular proportions across different stages of the cell cycle in the three groups (H9, *SNW1*$^{+/-}$ (#1-2 and #2-4) NPCs). Cells were divided into G0/1, S, and G2/M phases by DAPI and EdU co-staining. (I) Comparison of NPC proportions across three cell lines in G0/1, S, G2/M phases. Data was shown as mean ± SEM. One-way ANOVA was used for differential analysis, and no significant difference (n=3 batches for each cell line).

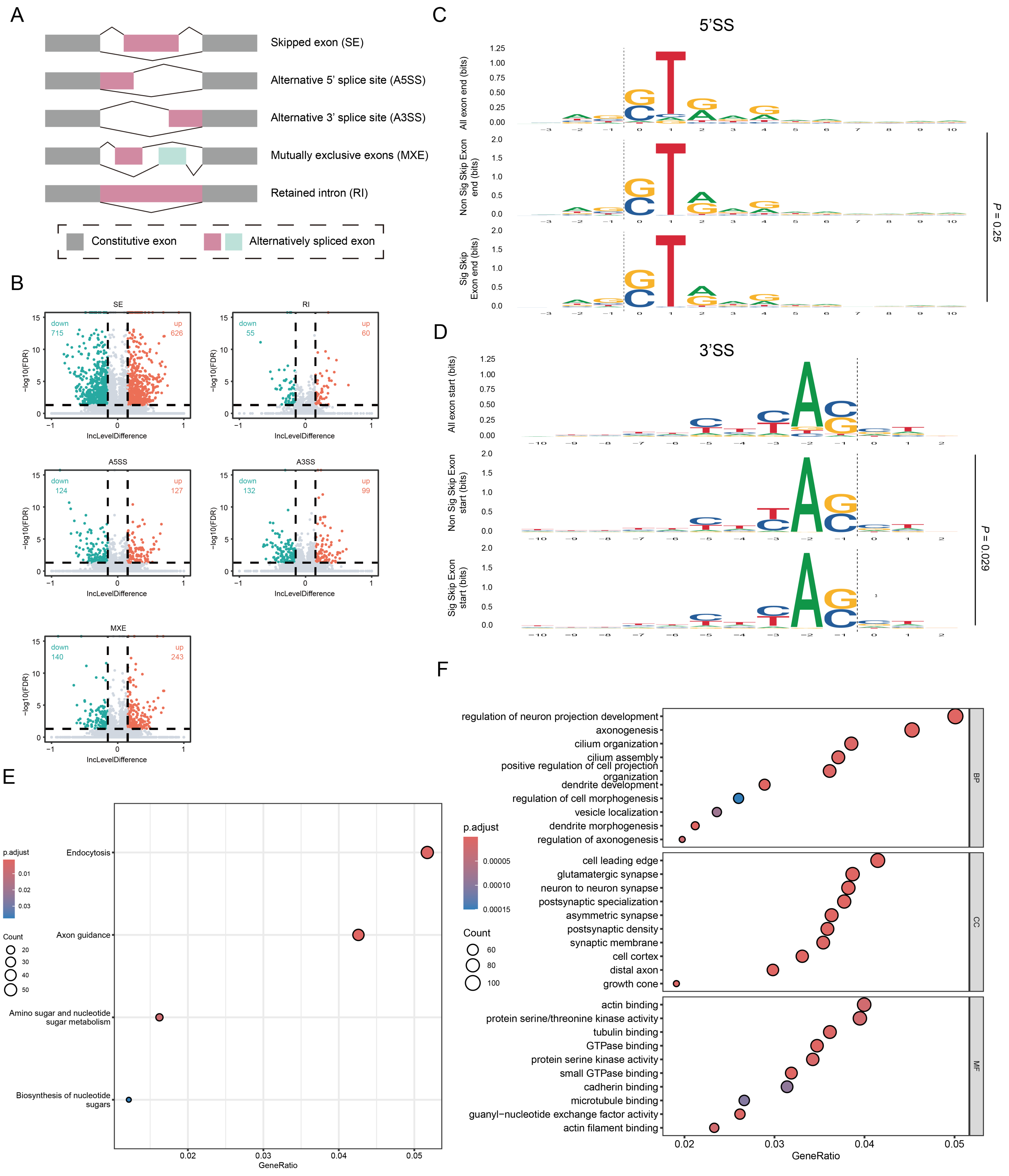

Supplementary Figure 7 Characterization of alternative splicing events in *SNW1*$^{+/-}$ brain organoids.

(A) The Schematic of five different types of alternative splicing (AS) events. Note: A5SS, alternative 5′ splice site; SE, skipped exon; MXE, mutually exclusive exon; RI, retained intron; A3SS, alternative 3′ splice site. (B) Volcano plots depicting differentially spliced events in five alternative splicing types. (C-D) Splice-site strength analysis of 5'SS and 3'SS in all exons, non-significant SE events, and significant SE events identified using rMATS. (E) KEGG enrichment analysis of exon skipping genes (ESGs) between wildtype and SNW1$^{+/-}$ (#1-2) brain organoids. (F) GO term enrichment analysis of exon skipping genes (ESGs) between wildtype and SNW1$^{+/-}$ (#1-2) brain organoids.

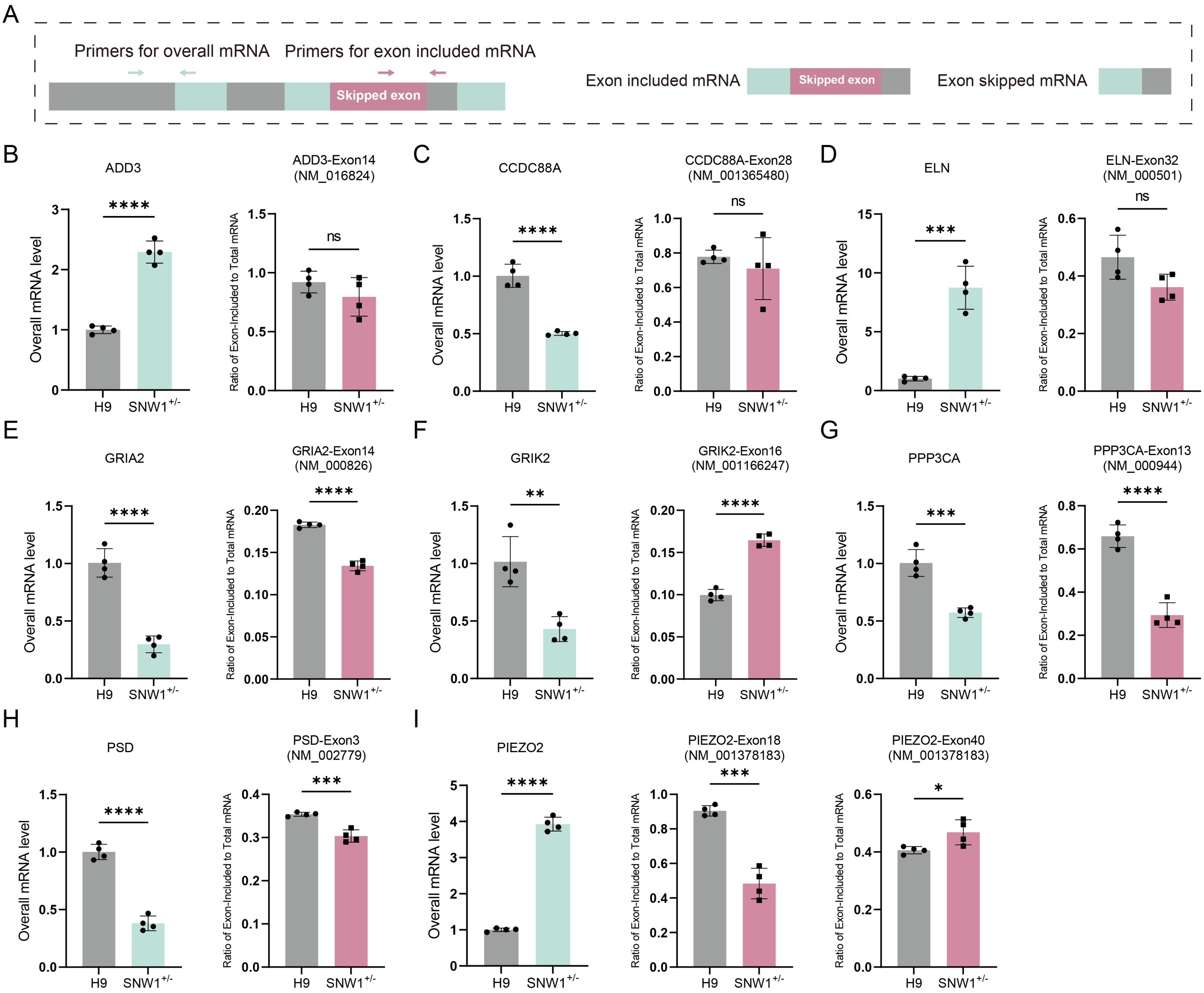

Supplementary Figure 8 qPCR validations of SE events in wildtype and SNW1$^{+/-}$ (#1-2) brain organoids.

(A) Primers used for detection of relative level of overall mRNA (sky blue) and exon-included RNA (pink). (B-I) Validation of significant exon skipping events by qPCR using *GAPDH* as reference gene in wildtype and SNW1$^{+/-}$ (#1-2) brain organoids. Relative level of overall mRNA (sky blue); ratio of Exon-included mRNA to total mRNA (pink). Data are presented as mean ± SEM. **P* < 0.05, ***P* < 0.01, ****P* < 0.001, *****P* < 0.0001, ns, no significance.

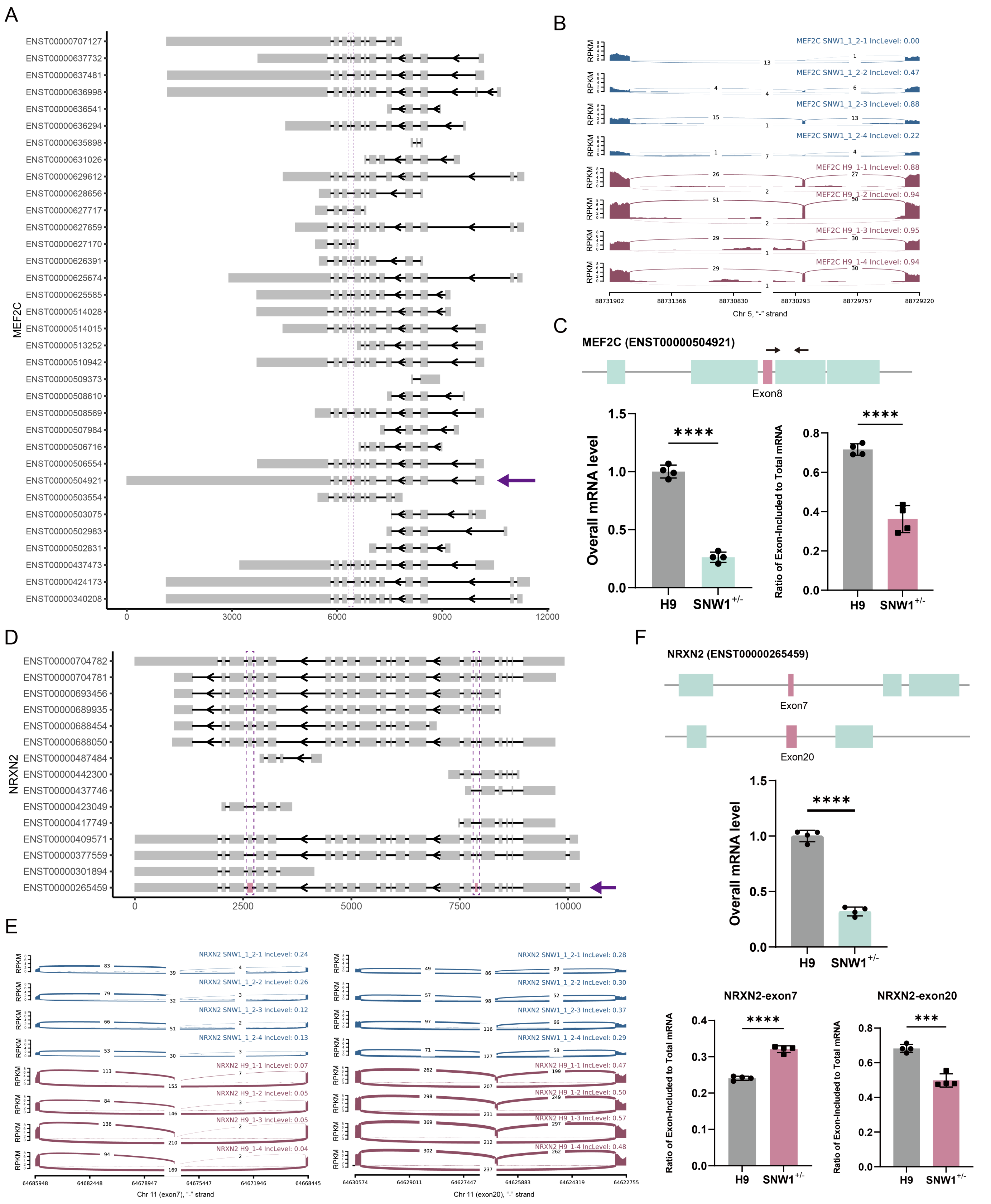

Supplementary Figure 9 Splicing alterations in *MEF2C* and *NRXN2* transcripts in *SNW1*$^{+/-}$ hCOs.

(A) Schematic representation of human *MEF2C* transcripts isoforms. The purple arrows indicate the MANE-selected canonical transcripts. The identified exons in the rMATS analysis are marked in pink. (B) Sashimi plots of read density of *MEF2C* transcript in 4 *SNW1*$^{+/-}$ (#1-2) and 4 control brain organoids revealed that *SNW1*$^{+/-}$ hCOs exhibited a tendency to skip exon 8, whereas the wild-type tended to retain this exon. (C) qPCR validations of SE events of *MEF2C* in wildtype and *SNW1*$^{+/-}$ (#1-2)brain organoids. Top: Schematic diagrams of *MEF2C* transcript. The pink boxes represent the skipped exons. Bottom: validation of significant exon skipping events by qPCR using *GAPDH* as reference gene. (D) Schematic

representation of human *NRXN2* transcripts isoforms. The purple arrows indicate the MANE-selected canonical transcripts. The identified exons in the rMATS analysis are marked in pink. (E) Sashimi plots of read density of *NRXN2* transcript in 4 *SNW1*$^{+/-}$ (#1-2) and 4 control brain organoids revealed that *SNW1*$^{+/-}$ hCOs exhibited a tendency to retention of exon 7 and skip exon 20, whereas the wild-type demonstrated the opposite pattern. (F) qPCR validations of SE events of *NRXN2* in wildtype and *SNW1*$^{+/-}$ (#1-2) brain organoids. Top: Schematic diagrams of *NRXN2* transcript. The pink boxes represent the skipped exons. Bottom: validation of significant exon skipping events by qPCR using *GAPDH* as reference gene. Relative level of overall mRNA (sky blue); ratio of Exon-included mRNA to total mRNA (pink). ***$P < 0.001$, ****$P < 0.0001$.

Supplementary Tables 1-5

Supplementary Table 1. P/LP variants not directly linked to the clinical phenotype of the patients.

Gene and transcript	Location	Nucleotide and amino acid changes	Genotype	Population frequency	Pathogenicity classification	Disease/Phenotype [Inheritance pattern]	Origin
PDZD7 NM_001195263.2	10:102770325	c.2320_2321insA p.Ser774Alafs*34	Het	-	LP	1. Deafness autosomal recessive 57; DFNB57[AR] 2. Usher syndrome type IIC; USH2C[AR] 3. Retinal disease in Usher syndrome type IIA; USH2A[AR]	**DE NOVO**
TNFRSF13B NM_012452.3	17:16852187	c.310T>C p.Cys104Arg	Het	0.0054	LP	1. Immunodeficiency common variable 2; CVID2[AR] 2. Immunoglobulin A deficiency 2; IGAD2[AR]	**DE NOVO**
PINK1 NM_032409.3	1:20972133	c.1040T>C p.Leu347Pro	Het	0.0003	P	Parkinson disease 6 early onset; PARK6[AR]	**DE NOVO**
FUT2 NM_000511.6	19:49206817	c.604C>T p.Arg202Ter	Het	0.0060	LP	1. Bombay phenotype (with digenic effect); Bombay[AR] 2. Resistance to Norwalk virus/vitamin B12; B12R[AR]	**DE NOVO**
ALOX12B NM_001139.3	17:7984461	c.397A>G p.Arg133Gly	Het	-	LP	Ichthyosis congenital autosomal recessive 2; CIEAR2[AR]	**DE NOVO**
CCBE1 NM_133459.3	18:57134013	c.511G>A p.Gly171Arg	Het	-	LP	Hennekam Lymphangiectasia-Lymphedema Syndrome Type 1; HKLLS1[AR]	**DE NOVO**
COL18A1 M_001379500.1	21:46924328	c.2728-2A>G NA	Het	-	P	Knobloch syndrome 1; KNO1[AR]	**DE NOVO**
CPLANE1 M_001384732.1	5:37206436	c.3012T>A p.Tyr1004*	Het	-	P	Joubert syndrome; JS[AR]	**DE NOVO**
ABCA12 NM_173076.3	2:215896579	c.1027G>T p.Gly343*	Het	-	LP	1. Ichthyosis congenital autosomal recessive 4A; CIEAR4A[AR] 2. Ichthyosis congenital autosomal recessive 4B (harlequin); CIEAR4B[AR]	**DE NOVO**
CERKL NM_201548.3	2:182402945	c.1561_1564dup p.(Y522Sfs*19)	Het	0.0001	LP	Retinitis Pigmentosa 26; RP26[AR]	Maternal
QARS1 NM_005051.3	3:49137404	c.1284_1285delAG p.(D430Qfs*16)	Het	0	P	Microcephaly, Seizures, and Cortical and Cerebellar Atrophy; MSCCA[AR]	Maternal

MMUT<br>NM_000255.4	6:49409685	c.1677-1G>A<br>-	Het	0.0004	P	Methylmalonic Aciduria, mut type; MMA mut[AR]	Paternal
DGAT1<br>*NM_012079.4*	8:145550166	c.134(exon1)delA<br>p.(D45Afs*22)	Het	-	LP	Diarrhea 7, Protein-Losing Enteropathy Type; DIAR7[AR]	Maternal
TG<br>NM_003235.4	8:134034365	c.7006C>T<br>p.Arg2336*	Het	0.0002	P	1. Thyroid Hormone Deficiency Type 3; TDH3[AR]<br>2. Autoimmune Thyroid Disease Susceptibility 3; AITD3[-]	Paternal

Note: The genome version is hg19; the population frequency is the highest allele frequency from the 1000 Genomes, ExAC, and gnomAD databases; AD: autosomal dominant, AR: autosomal recessive

Supplementary Table 2. Clinical features of the patients.

	Individual 1[A]	Individual 2[B]	Individual 3[C]	Individual 4[D]	Individual 5[E]	Individual 6[F]	Individual 7[G]	Individual 8[H]	Individual 9[I]
Mutation cDNA	c.182_187del	c.691_689delins GGT	c.330+2T>C	c.614A>C	c.-2_1del	c.187G>C	c.1235_1236ins A	c.426+1G>A	c.426+1G>T
Gender	F	F	F	M	F	M	M	F	F
Microcephaly	yes	yes	yes	yes	yes	yes	NA	yes	yes, severe
At birth									
Gestational age, weeks	spontaneus birth at term, 40 weeks (+2days)	40	40	NA	NA	38	NA	39	full term
Length cm	51	50	48.5	48	NA	NA	NA	49	45.7
Weight g	150	3100	3420	2730	NA	2950	NA	3200	2608
OFC cm (Z score)	28 (-4.6SD)	29 (-3.93SD)	32 (-1.9SD)	33 (-1.27SD)	NA	NA	NA	30 (-2.85SD)	NA
Abnormal muscolar tone	hypotonic at 2 months of age	no	no	no	NA	no	NA	hypotonia	NA
At last evaluation									
Age	20	8 y	18 y 7m	7 y	24 y	3 y 9 m	19w5d	9 y 4 m	9 y
Hight cm (Z score)	150 (-2.04SD)	105 (-4.0SD)	152 (-1.7SD)	116 (-1.5 SD)	157 (-1.19 SD)	101	NA	130 (-0.23SD)	122.2 (-3.41SD)
Weight kg (Z score)	27 kg (BMI 12)	15 (-3.2 SD)	40 (-2.6 SD)	20 (-1.5 SD)	54.1 (-0.42 SD)	13 (-1.74 SD)	NA	20 (-2.1 SD)	24.9 (-2.41SD)
OFC cm (Z score)	45 (-8.8SD)	39 (-10.3SD)	47 (-6.9SD)	48 (-3.5SD)	49.3 (-4.52 SD)	44.5 (-3.6 SD)	NA	44 (-6.5SD)	39.4 (-6.93SD)
Feeding difficulties	yes, fed by PEG	yes	Dyspagia; PEG.	yes	no	no	NA	yes	poor swallow

	Individual 1	Individual 2	Individual 3	Individual 4	Individual 5	Individual 6	Individual 7	Individual 8	Individual 9
Neuropsichiatric manifestations									
Seizures (onset age)	focal-onset clonic and focal to bilateral tonic clonic seizures (3rd month of life)	generalized seizures, 6 months old	Partial seizures (onset at 2 months), then developmental and epileptic encephalopathy.	tonico-clonic seizures during fever, 12 months old	no	FS, 7 months old; CPS, 2 years old; recently, CPS, 2 times/month	NA	infantile spasm (4 months)	yes, prior to 8 m
DD/ID (IQ)	yes (IQ<50)	yes (NA)	yes (profound)	yes	yes	yes	NA	yes (30)	yes, prior to 8 m
Language delay	no speech	speech absent	speech absent	yes	yes	speech delay	NA	speech absent	lack of expressive language
Motor delay (age at walking)	severe, no walking (hip dislocation right side, left subluxation)	yes, could not walk	yes, spastic tetraplegia	no	no	no	NA	yes, could not walk	uses gait trainer and wheelchair, walks with assistance
Indipendent toileting delay (age)	no	yes	no	NA	yes	yes	NA	yes	No
Stereotypic movements	dyskinetic cerebral palsy	yes	no	no	no	yes	NA	no	hang wringing, biting fingers
Abnormal behaviour	no	no	no	yes	yes	yes	NA	no	no
ASD	no	no	no	yes	no	no	NA	no	no
Sleep disturbancies (specify)	no	no	alteration sleep-wake rithm	no	no	no	NA	no	no

	Individual 1	Individual 2	Individual 3	Individual 4	Individual 5	Individual 6	Individual 7	Individual 8	Individual 9
Brain MRI									
Corpus callosum hypoplasia	NA	NA	yes	NA	Focal defect of the middle third of the cerebral sickle	NA	yes	yes	no
White matter abnormalities	NA	NA	no	NA	no	NA	No	no	no
Other	only MRI at 2 months of age showed hypolastic pachygiric cerebrum and atrophic cerebellum	NA	Brain atrophy; Dandy-Walker malformation	NA	Discrete tortuosity of intracranial arterial vessels	NA	Dandy-Walker malformation,bilateral ventriculomegaly, enlarged CM, dilated 4th ventricle, hypoplastic corpus callosum	Brain atrophy	cerebral dysgenesis noted at MRI prior to 8 mo, (2/11/2020): a small Rathke's cleft cyst, subtotal opacification of the mastoid air cells bilaterally
Ocular manifestations									
Exoforia	Strabismus divergens with pendular nystagmus	no	no	no	During first years of lifemarked	no	NA	no	No
Other	severe visual impairment	no	Cortical visual impairment	no		no	NA	no	myopia

	Individual 1	Individual 2	Individual 3	Individual 4	Individual 5	Individual 6	Individual 7	Individual 8	Individual 9
EEG									
Sleep	normal	NA	Multifocal epileptiform discharges	NA	NA	Spike and spike-slow waves in the right temporal and occipital areas	NA	Multifocal epileptiform discharges	no
Awake	normal	NA	Multifocal epileptiform discharges; EEG with bilateral mostly anterior spike-wave complexes	NA	NA	Spike and spike-slow waves in the right temporal and occipital areas	NA	Multifocal epileptiform discharges	recurrent left and right independent spikes and sharp waves seen in the posterior quadrants of each hemisphere more than the anterior quadrant.
Skeletal manifestations									
Scoliosis	thoracolumbar	no	no	no	no	no	NA	yes	No
Kyphosis	mild	no	no	no	no	no	NA	no	no
Other	hip dislocation	no	osteopenia	no		no	NA	no	no

Note: The source units for the individuals listed in this table are provided below. **A**, Institute of Human Genetics, University of Leipzig Medical Center, Leipzig, Germany; Kleinwachau Epilepsy Center, Radeberg, Germany. **B**, Department of Medical Genetics, Hunan Provincial Maternal and Child Health Care Hospital, Changsha, China; The Affiliated Children's Hospital Of Xiangya School of Medicine,Central South University (Hunan children's hospital); Clinical Medical Research Center For Hereditary Birth Defects and Rare Diseases In Hunan Province; National Health Commission Key Laboratory for Birth Defect Research and Prevention, Hunan Provincial Maternal and Child Health Care

Hospital, Changsha, China; Chigene (Beijing) Translational Medical Research Center Co. Ltd, Beijing, China. **C**, Institute for Maternal and Child Health, IRCCS "Burlo Garofolo", Trieste, Italy; Institute for Maternal and Child Health, IRCCS "Burlo Garofolo", Trieste, Italy. **D**, Department of Genetics, APHP-Robert Debré University Hospital, Paris, France; INSERM UMR1141, Neurodiderot, University of Paris Cité, Paris, France. **E**, Department of Clinical and Molecular Genetics, Vall d'Hebron Barcelona Hospital Campus, Barcelona, Spain; Medicine Genetics Group, Vall Hebron Research Institute, Vall d'Hebron Barcelona Hospital Campus, Autonomous University of Barcelona, Barcelona, Spain. **F** Department of Neurology, Institute of Neuroscience, Key Laboratory of Neurogenetics and Channelopathies of Guangdong Province and the Ministry of Education of China, The Second Affiliated Hospital, Guangzhou Medical University, Guangzhou, China; **G**, Department of Pathology and Laboratory Medicine, University of California, Los Angeles, CA; Department of Obstetrics and Gynecology, David Geffen School of Medicine at UCLA, Los Angeles, CA, USA; Comprehensive Materna Fetal Medicine Center, Thousand Oaks, CA, USA. **H**, Department of Neurology, Jiangxi Provincial Children's Hospital, Nanchang, China. **I**, Department of Human and Molecular Genetics, VCU Health School of Medicine, VA, USA; Department of Pediatrics Genetics, VCU Health CHOR, VA, USA.

Supplementary Table 3. Prediction Results of SNW1 Splicing Variants.

Mutation cDNA	Genomic Variant (GRCh38)	MAXENT	FlyBase Splice Site	ESEfinder	spliceAI score	HSF
c.330+2T>C	chr14:77751317 A>G	7.46 --> -0.3	0.98 --> N.A.	8.8229 --> N.A.	0.21	Broken WT donor Site
c.426+1G>A	chr14:77738965 C>T	8.77 --> 0.59	0.92 --> N.A.	8.3864 --> N.A.	1	Broken WT donor Site
c.426+1G>T	chr14:77738965 C>A	8.77 --> 0.27	0.92 --> N.A.	8.3864 --> N.A.	1	Broken WT donor Site

Supplementary Table 4. Summary of functional impact of SNW1 variants.

Mutation cDNA	Protein change	Mutation Type	Functional Consequence
c.330+2T>C	D57_K110del/V90_K110del	Splice donor site mutation	Causes partial or complete skipping of exon 3, generating two aberrant transcripts. The D57_K110del isoform exhibits loss of interaction with PPIL1.
c.426+1G>A	V111_E142 del	Splice donor site mutation	Results in skipping of exon 4.
c.426+1G>T	V111_E142 del	Splice donor site mutation	Results in skipping of exon 4.
c.1235_1236insA	F412Lfs*17	Frameshift mutation	Subject to NMD. The escape isoform fails to localize to the nucleus and leads to cytoplasmic retention of PPIL1.
c.182_187del	G61_G62del	PPIL1 interaction site mutation	Loss of interaction with PPIL1; reduced protein expression.
c.187G>C	A63P	PPIL1 interaction site mutation	Loss of interaction with PPIL1.
c.614A>C	D205A	PRPF8 interaction site mutation	Attenuates SNW1 interaction with PRPF8; decreases protein expression.
c.691_689delinsGGT	M230_H231delinsRY	PLRG1 interaction site mutation	Enhances SNW1–PLRG1 interaction; increases protein expression.
c.-2_1del	\	Start codon loss	Abolished protein expression.

Supplementary Table 5. Antibodies and manufacturers

Antibody	Company	Catalog No.	Concentrations
Flag M2	Sigma-Aldrich	F1804	1:10,000
HA (cells)	CST	#3724	1:1000
HA (Fly)	BioLegend	901501	1:1000
GAPDH	CST	#2118	1:1000
SKIP (SNW1)	Santa Cruz	sc-393856	1:1,000
mCherry	CST	#43590	1:1,000
Deadpan	Abcam	ab195173	1:1,000
pHH3 (Fly)	Sigma Aldrich	06-570	1:500
Donkey anti-rat Alexa fluor 647	Jackson ImmunoResearch Laboratories Inc.	712-605-153	1:500
Donkey anti-rabbit Rhodamine RedX	Jackson ImmunoResearch Laboratories Inc.	711-295-152	1:500
Donkey anti-mouse Rhodamine RedX	Jackson ImmunoResearch Laboratories Inc.	715-295-151	1:500
DAPI	Beyotime	C1005	1:1000

Supplementary Table 6. Primers used in this study.

Primer type	ID	forward sequence	reverse sequence	Species	description
PCR	SNW1-c.182_187_del	GAGATGCTTTTCCAGAGATCCATGTGGC	GGAAAAGCATCTCCAAAATCCTCTAATAACCG	human	primers were used for PCR-mediated site mutation
	SNW1-c.187G>C	GAGGTCCTTTTCCAGAGATCCATGTGGCCCAG	GGAAAAGGACCTCCATCTCCAAAATCCTCTAATAACCG	human	primers were used for PCR-mediated site mutation
	SNW1-Ex3-KO	GGTTATTAGAGGTCATTTATAGCAAATACACTGACCTGG	TAAATGACCTCTAATAACCGAGGTATCCAGCCTTTC	human	primers were used for PCR-mediated site mutation
	SNW1-Ex3(63)-KO	GGCCATTCAGGTCATTTATAGCAAATACACTGACC	AATGACCTGAATGGCCAGCGCATTCGACAT	human	primers were used for PCR-mediated site mutation
	SNW1-c.614A>C	GAAAGCTCCAATGGAGCCTCCAAGG	CATTGGAGCTTTCTGCATTTCTACCATCCG	human	primers were used for PCR-mediated site mutation
	SNW1-c.689-691_delinsGGT	GCCGAAAGATGACTGTAAAGGAACAACAAGAGTGGAAGATTC	CTTTACAGTCATCTTTCGGCTAGGAGAATACCTGACAGGC	human	primers were used for PCR-mediated site mutation
	SNW1-c.1235_1236insA	CTCTTACAACCAATCCAAGGGTATGGACAG	GATTGGTTGTAAGAGCCTTTGGTCATACTGAAC	human	primers were used for PCR-mediated site mutation
	SNW1-Ex4-KO	AAGACAAGATAACAGAAAAGACAAGAGTAGCCTTAG	GTTATCTTGTCTTTTGACTGTCCTTGTCGAGCAA	human	primers were used for PCR-mediated site mutation
	PPIL1_pCMV-HA-N	CATGGAGGCCCGAATGGCGGCAATTCCCC	CGCGGCCGCGCTACCCAGAAGGGTATG	human	primers were used for *PPIL1* overexpression in pCMV-HA-N
	PLRG1_pCMV-HA-N	CCATGGAGGCCCGAATGGTCGAGGAGGTACAGA	CCGCGGCCGCGGCCACATTCATTAAAATCTCT	human	primers were used for *PLRG1* overexpression in pCMV-HA-N
	PRPF8_pCMV-HA-N	CATGGAGGCCCGAATGGCCGGAGTGTTTCCT	CGCGGCCGCGTCAGGCATACAGGTCCTC	human	primers were used for *PRPF8* overexpression in pmCherry-N1
	PPIL1_mcherry	ACCGGACTCAGATCATGGCGGCAATTCCCC	CCCGCGGCCCAGAAGGGTATGCCTT	human	primers were used for *PPIL1* overexpression in pCMV-HA-N

	PLRG1_mcherry	ccggactcagatctATGGTCGAGGAGGTACAGAA	ccgggcccgcggtaTTAAAATCTCTTTCTCTTGAT	human	primers were used for *PLRG1* overexpression in pmCherry-N1
	SNW1-c.330+2T>C_minigene	GTCCCCAACCACCCTATCAA	TGCAAAGACAGTAGACAGGC	human	Primers for constructing SNW1 c.330+2T>C minigene reporter vector
	SNW1-c.330+2T>C_Mutation	AGACAAGGCAATCCTCTCATGTTTTCCCTGAATGATA	GAGGATTGCCTTGTCTTTTGACTGTCCTTGTCGAG	human	Primers were used for PCR-mediated site mutation
	SNW1-c.426+1G/T_minigene	CGGGATCCGCTGAGAGGGCATGTTGA	CGACGCGTGCAAAGACAGTAGACAGGC	human	Primers for constructing SNW1 c.426+1G/T minigene reporter vector
	SNW1-c.426+1G>A_Mutation	AGAGATAACTGGGAATTGATATATTCGATGACTATTTACATCC	CAATTCCCAGTTATCTCTTTAATAGCTTCTTCATCGGG	human	Primers were used for PCR-mediated site mutation
	SNW1-c.426+1G>T_Mutation	AGAGTTAACTGGGAATTGATATATTCGATGACTATTTACATCC	CAATTCCCAGTTAACTCTTTAATAGCTTCTTCATCGGG	human	Primers were used for PCR-mediated site mutation
RT-PCR	pCAS2-RT	CTGACCCTGCTGACCCTCCT	TTGCTGAGAAGGCGTGGTAGAG	human	Primers specific to the pCAS2 vector
	SNW1_c.1235insA_NMD	TACCCCAACCACAGTGAGGA	AGTTGCACCAAGCAAGAGGT	human	Primers specific to the NMD vector
sgRNA	SNW1-exon5-sgRNA1	CACCGACATCTGAAATGGCTAATG	AAACCATTAGCCATTTCAGATGTC	human	guide RNAs targeting the introns flanking exon 4 and exon 5 to generate SNW1 knockout cell lines in H9 hESCs
	SNW1-exon5-sgRNA2	CACCgTGTCTGCAAAGACAGTAGAC	AAACGTCTACTGTCTTTGCAGACAC	human	guide RNAs targeting the introns flanking exon 4 and exon 5 to generate SNW1 knockout cell lines in H9 hESCs
RT-qPCR	rp49 qPCR	ATGACCATCCGCCCAGCATACA	CGTAACCGATGTTGGGCATCAGATACT	Drosophila	Primers for overall mRNA
	Bx42 qPCR	GCGCAATATCCACTAGGTCTG	GTCGTCTCCATCACCGTTTC	Drosophila	Primers for overall mRNA

Actin qPCR	TGACGTGGACATCCGCAAAG	CTGGAAGGTGGACAGCGAGG	human	Primers for overall mRNA
ADD3-overall mRNA	CCAGCCAAGGCGTGATTAC	TGAAGTCTTGTCGTAGATCAGGA	human	Primers for overall mRNA
CCDC88A-overall mRNA	ATGCCTCACTTAGAATGCACAA	AGACATTTGGCAACGACATCA	human	Primers for overall mRNA
ELN-overall mRNA	GCAGGAGTTAAGCCCAAGG	TGTAGGGCAGTCCATAGCCA	human	Primers for overall mRNA
FBN1-overall mRNA	GCGGAAATCAGTGTATTGTCCC	CAGTGTTGTATGGATCTGGAGC	human	Primers for overall mRNA
GRIK2-overall mRNA	TTCAGGCGCACCGTTAAACT	GCTCCCATTGGGCCAGATT	human	Primers for overall mRNA
PPP3CA-overall mRNA	GCGCATCTTATGAAGGAGGGA	TGACTGGCGCATCAATATCCA	human	Primers for overall mRNA
PSD-overall mRNA	AGCCGGATGCCTCTCAAGT	CCCGGTGTGACTCCAGGAT	human	Primers for overall mRNA
GRIA2-overall mRNA	CATTCAGATGAGACCCGACCT	GGTATGCAAACTTGTCCCATTGA	human	Primers for overall mRNA
NRXN2-overall mRNA	CAGCACGAGGATGGATCGC	GCCCACGTTAAAGATCACCCC	human	Primers for overall mRNA
PIEZO2-overall mRNA	ACGACGATGCAAGGACATACG	GCTCACCAACGTGATGTGG	human	Primers for overall mRNA
ADD3-Exon-included	CACCGCAAAATGTCCCTGAA	CTACCATGACAGGCACTTCCA	human	Primers for exon included mRNA
CCDC88A-Exon-included	GTCCACAAGGTGTTAGTGATGA	TCTGCCACTATCAAGGCTGG	human	Primers for exon included mRNA
ELN-Exon-included	CTAAATACGGTGCTGCTGGC	ATGGGAGACAATCCGAAGCC	human	Primers for exon included mRNA
FBN1-Exon-included	ACAATCGGGAAGGGTACTGC	GCATTCCGATTTGGTGACGG	human	Primers for exon included mRNA
GRIK2-Exon-included	CCTCCCTATTTTGGAGTCAGTT	TACCATGGCACTACAGAAGGAC	human	Primers for exon included mRNA
PPP3CA-Exon-included	TGTTGAGGCTATTGAGGCTGA	ATTCGGTCTAAGCCCTTGGC	human	Primers for exon included mRNA
PSD-Exon-included	CCCGGGAAGTCTCCTCTCAT	CCTGCTCTGGGGGCTTAG	human	Primers for exon included mRNA
GRIA2-Exon-included-1	ATGGCATCGCAACACCTAAAG	GAGTCCTTGGCTCCACATTCA	human	Primers for exon included mRNA
GRIA2-Exon-included-2	CATTCAGATGAGACCCGACCT	GGTATGCAAACTTGTCCCATTGA	human	Primers for exon included mRNA
NRXN2-Exon-included-1	CCCAACGCCATAGTAAGCGA	CCAGGCGCTCGTTATCAAAG	human	Primers for exon included mRNA

NRXN2-Exon-included-2	CCCTTGTGGAACCCGTCAAT	ACCATAGCGTGTCCAATCCC	human	Primers for exon included mRNA
PIEZO2-Exon-included-1	ACCGGTTCCTTGAACTCACA	GCGACCATTGACTTTGGCAT	human	Primers for exon included mRNA
PIEZO2-Exon-included-2	ACACCATTGACGAGCATCCC	GGACTGCCTTGAGAACAGCA	human	Primers for exon included mRNA

Supplemental Methods

Minigene reporter assay

The pCAS2 construct spanning *SNW1* exons 3, 4 and 5, along with 262 bp of upstream and 145 bp of downstream intronic sequences, was used as wild-type (WT) template for generating the *SNW1* c.330+2 T>C minigene (1). The mutations were inserted as described above. The pCAS2 vector was linearized using MluI (NEB, R3198) and BamHI (NEB, R3136) The WT and mutated sequences were cloned into the vector using One Step Cloning Kit (Yeasen, 10911). The constructs were validated with Sanger sequencing. HEK293T cells were transfected with the mutant and WT constructs in 12-well plates at a concentration of 100 ng/well. After 48 hours, RNA was extracted (Accurate Biology, AG21023) and reverse transcribed into cDNA (Accurate Biology, AG11728) according to the manufacturer's instructions. To detect the isoform content of the cDNA, primers specific to the pCAS2 vector were used in RT-PCR. The identified isoforms were then cloned into T-vectors and verified by Sanger sequencing.

For the NMD minigene reporter, exons 11 to 14 were inserted into the pEGFP-C1 vector and co-transfected into cells alongside the pmCherry-C1 vector as an internal control. An NMD analysis was carried out by transiently transfecting plasmids into HEK293T cells. Total protein was extracted for Western blotting analysis. Cycloheximide (CHX) (100 μg/ml) (GLPBIO, CA, USA) was added after 24 h of incubation, and total RNA was extracted 5 h later for qPCR analysis.

Plasmid constructs

The cDNA sequence of *SNW1* (NM_012245.3) was obtained from the cDNA library and inserted into pcDNA3.1-3xFlag-C and pEGFP-C3 using One Step Cloning Kit. We performed mutagenesis on the wild-type (WT) cDNA using Q5 PCR to generate the point mutation found in microcephaly patients. The cDNA sequences of *PPIL1* (NM_016059.4), *PLRG1* (NM_002669.4), and *PRPF8* (NM_006445.4) were also cloned into pCMV-HA-N and pmCherry-C1. Primer sequences can be found in the Supplementary Table 5. The constructs were confirmed by Sanger sequencing.

Generation of human embryonic stem cell-derived NPCs

Human embryonic stem cell-derived neural progenitor cells (NPCs) were established from H9 human embryonic stem cells (hESCs) and SNW1-knockout (SNW1-KO) cell lines using the Dual-SMAD inhibition method. Briefly, single-cell suspensions were generated by treating the cells with EDTA and Accutase. For initial plating, 300,000 cells were seeded into each well of a Matrigel-coated, tissue culture-treated 24-well plate in mTeSR medium supplemented with 10 μM ROCK inhibitor (Selleck). After 24 hours, neural induction was performed if the cells have reached 95~100% confluence. To induce NPCs, the medium was replaced with 2D neural induction medium containing a 1:1 mixture of DMEM/F12 and Neurobasal (Invitrogen, #21103049), 0.5×N2 supplement, 1×B27 supplement with vitamin A (Invitrogen, #17504044), 2.5 ng/mL insulin

(Sigma, #I9278-5ML), 1% P/S, 1×Glutamax, 1×MEM-NEAA, 1 μM Dorsomorphin (Selleckchem, #S1067) and 10 μM SB431542 (Selleckchem, #S7840). Cells were then fed with fresh 2D neural induction medium every day for 12 days to obtain NPCs. Confluent NPCs, harvested on day 12, were used for subsequent analyses.

Immunoprecipitation assay and western blotting

HEK293T cells were seeded in 6 cm dishes and transfected with the indicated plasmids using Lipo8000 (Beyotime, C0533) after 24 hours. After 48 hours of transfection, the cells were lysed using RIPA buffer with protease inhibitor cocktail (Epizyme Biotech, GRF101), and the lysates were centrifuged at 12,000 rpm for 15 minutes at 4°C to obtain the supernatant. Ten percent of the supernatant was mixed with 5× SDS-loading buffer (Epizyme Biotech, LT101) and heated at 95°C for 10 minutes to prepare the total lysates. The remaining supernatant was incubated with pre-equilibrated anti-Flag magnetic beads (Selleck, B26101) at 4°C overnight. The beads were washed four times with 0.5% PBST and eluted with 50 μl of 1× SDS-loading buffer. The total lysates and immunoprecipitates were separated by SDS-PAGE and analyzed by immunoblotting. The primary antibody used in this study include mouse anti-Flag M2 (1:10,000, Sigma-Aldrich, F1804), rabbit anti-HA (1:1000, CST, #3724), rabbit anti-GAPDH (1:1000, CST, #2118), mouse anti-SKIP (1:1,000, Santa Cruz, sc-393856), and rabbit anti-mCherry (1:1000, CST, #43590).

Immunofluorescent staining and Immunohistochemistry

To visualize SNW1 and other target proteins, cells cultured on coverslips can be transfected with plasmid encoding green (SNW1) or red (other proteins) fluorescent fusion proteins under an appropriate promoter. Medium was removed and cells were then rinsed with 1×PBS for 2 times. Cells were fixed with 4% paraformaldehyde (PFA) for 15 min at room temperature, followed by 3×10 min washing with 1×PBS by gentle rocking on a shaker. Stain cells with 4'6-diamidino-20-phenylindole (DAPI; Beyotime, C1005) and allowed to incubate for 5 min at RT on the shaker. Cells were then washed and mounted onto the slide with drops of anti-fade solution (Beyotime, P0126) between, then seal the edges of coverslips with nail polish. Slides can be stored for 1–2 weeks at 4 °C in the dark before signal deterioration.

Wandering third-instar larvae were selected for by clearance of blue food from the gut and extruding spiracles. Third-instar brains were removed and fixed in 4% paraformaldehyde (PFA) in Phosphate Buffered Saline + 0.3% Triton (PBST), followed by three 5-minute washes in PBST. Brains were then washed with PBST + 1% bovine serum albumin (PBSTB) twice for 30 minutes, with a subsequent 30-minute wash with PBSTB plus 5% normal donkey serum. The following primary antibodies were added and incubated in PBSTB overnight at 4°C: rat anti-Deadpan (neural stem cells, 1:1000, Abcam, ab195173), mouse anti-HA.11 (1:1000, BioLegend, 901501), and rabbit anti-pHH3 (proliferation, 1:500, Sigma Aldrich 06-570). The next day, brains were washed three times with PBSTB for 20 minutes and then incubated in secondary antibodies: 1:500 Donkey anti-rat Alexa fluor 647 (Jackson ImmunoResearch Laboratories Inc., 712-605-153), 1:500 Donkey anti-rabbit Rhodamine RedX

(Jackson ImmunoResearch Laboratories Inc., 711-295-152), 1:500 Donkey anti-mouse Rhodamine RedX (Jackson ImmunoResearch Laboratories Inc., 715-295-151), DAPI (1:1000) for one hour. Finally, brains were washed 3 times with PBST and mounted in slow fade gold.

We performed sectioning and immunofluorescence staining on hCOs at day 45. This time point was selected because the volume phenotype of the organoids is highly stable, and both neural stem cell proliferation and neuronal differentiation are notably active at this stage. Cerebral organoids were fixed in 4% PFA at 4°C for a week, then sequentially transferred to 25% and 30% sucrose for dehydration overnight each, and embedded in O.C.T. (Sakura). The organoids were cryosectioned at 14 µm and 16 µm (Leica CM3050S). For antigen retrieval, the brain sections were incubated in boiling antigen retrieval buffer (1 mM EDTA, 5 mM Tris, pH 8.0) for 15 minutes and cooled to room temperature. The sections were blocked with 0.5% Triton X-100 and 4% bovine serum albumin (BSA) at room temperature for 1 hour. The sections were then incubated with primary antibodies overnight at 4°C, followed by incubation with secondary antibodies at room temperature for 1 hour.

Confocal Microscopy

Confocal images were taken by laser scanning confocal microscopy (Leica TCS SP8) in the sequential mode. Images were then processed with Leica Application Suite X 1.8.1.13759 software. For Drosophila studies, a singular brain lobe from each brain was imaged at 40X magnification at 0.7 zoom. Z stacks were set with Alexa fluor 647 channel looking at Deadpan signal with 2 µm slices through the whole brain. Images were taken at 1024x1024 frame size, 8 speed, line averaging of 2, z stacks of 2 µm size taken through the whole brain lobe.

Bulk RNA-sequencing (RNA-seq) and data analysis

Total RNA was extracted from hCOs (45-day-old) following the manual of RNeasy Plus Mini Kit (QIAGEN), yield and quality of RNA assessed by Qubit2.0 Fluorometer (Thermo Fisher Scientific) and Agilent 2100 Bioanalyzer (Agilent Technologies), respectively, enriched by poly-A capture. Paired-end libraries were prepared according to the manufacturer's protocols (TruSeq Stranded mRNA, Illumina) and sequenced using Illumina PE150 (Illumina). 20-24 million paired-end 150bp reads were collected for each sample. FASTQ files containing RNA-seq data from SNW1 and H9 samples were trimmed with trimm_galore (https://github.com/FelixKrueger/TrimGalore), aligned to reference human hg38 with GTF release 21 using STAR aligner with ENCODE standard options (2), and with 'end-to-end alignment type' checked as required by rMATS (3). The gene expression values (raw counts) were generated by featureCounts (4). Differential gene expression between sample groups was ascertained by R package DESeq2. The principal component analysis, volcano plot, heatmap and enrichment analysis were carried out by R packages DESeq2, ggplot2, pheatmap, clusterProfiler. Network analysis of enriched pathways was conducted on Metascape (https://metascape.org/gp/index.html#/main/step1).

rMATS computational analysis

Alternative splicing events were summarized by rMATS 3.2.5, with 'novel splice site detection' turned on. Heatmap representation of inclusion levels for each sample was plotted using R package pheatmap. All intron and exon positions for human were downloaded as one BED file from UCSC browser. Sequences from the human genome file were extracted using known start and end positions with bedtools getfasta function. Then the introns residing in alternative splicing events, were calculated in R ggplot2, with significance determined by FDR < 0.05 and the absolute value of inclusion level difference > 0.05. A one-sided Mann-Whitney test was performed to obtain p-value between each group. Sequences of 41bp in length (20bp extension toward each direction) around 50 and 30 consensus splice sites of all human introns as well as our splicing event introns were extracted and stacked as sequence logo plots using R package ggseqlogo.

Sashimi plot

Sashimi plot visualization of rMATS was generated with Python script rmats2sashimiplotm (https://github.com/Xinglab/rmats2sashimiplot), with sorted BAM files from STAR used as inputs. Event files contain selected events extracted from rMATS outputs. Plots for different splicing types were specified by -t argument.

RT-qPCR

Total RNA was extracted from cells or tissues with SteadyPure Quick RNA Extraction Kit (Accurate Biology, AG21023) and with PureLink RNA Mini Kit from ThermoFisher (12183018A) for Drosophila tissues. RNA was reverse transcribed into cDNA (Accurate Biology, AG11728 for human; biorad iScript cDNA Synthesis kit for Drosophila) following the manufacturer's instructions. RT-qPCR was performed using SYBR Green Master Mix (Accurate Biology, AG11701). Gene expression levels were normalized to GAPDH (huaman) and RpL32 (fly), and analyzed using the 2^-ΔΔCt method. Primers sequences used were listed in Supplementary Table 6.

References

1. Gaildrat P, Killian A, Martins A, Tournier I, Frebourg T, and Tosi M. Use of splicing reporter minigene assay to evaluate the effect on splicing of unclassified genetic variants. *Methods Mol Biol.* 2010;653:249-57.
2. Dobin A, Davis CA, Schlesinger F, Drenkow J, Zaleski C, Jha S, et al. STAR: ultrafast universal RNA-seq aligner. *Bioinformatics.* 2013;29(1):15-21.
3. Shen S, Park JW, Lu ZX, Lin L, Henry MD, Wu YN, et al. rMATS: robust and flexible detection of differential alternative splicing from replicate RNA-Seq data. *Proc Natl Acad Sci U S A.* 2014;111(51):E5593-601.
4. Liao Y, Smyth GK, and Shi W. featureCounts: an efficient general purpose program for assigning sequence reads to genomic features. *Bioinformatics.* 2014;30(7):923-30.
