# Supplementary material for: Mutations in the spliceosomal gene SNW1 cause neurodevelopment disorders with microcephaly
Source: J Clin Invest. 2025 Jul 3;135(18):e186119. doi: 10.1172/JCI186119 (PMC12435841; doi:10.1172/JCI186119)
Supplement: Unedited blot and gel images [file jci-135-186119-s228.pdf]

Full unedited blot/gel for Figure 1C

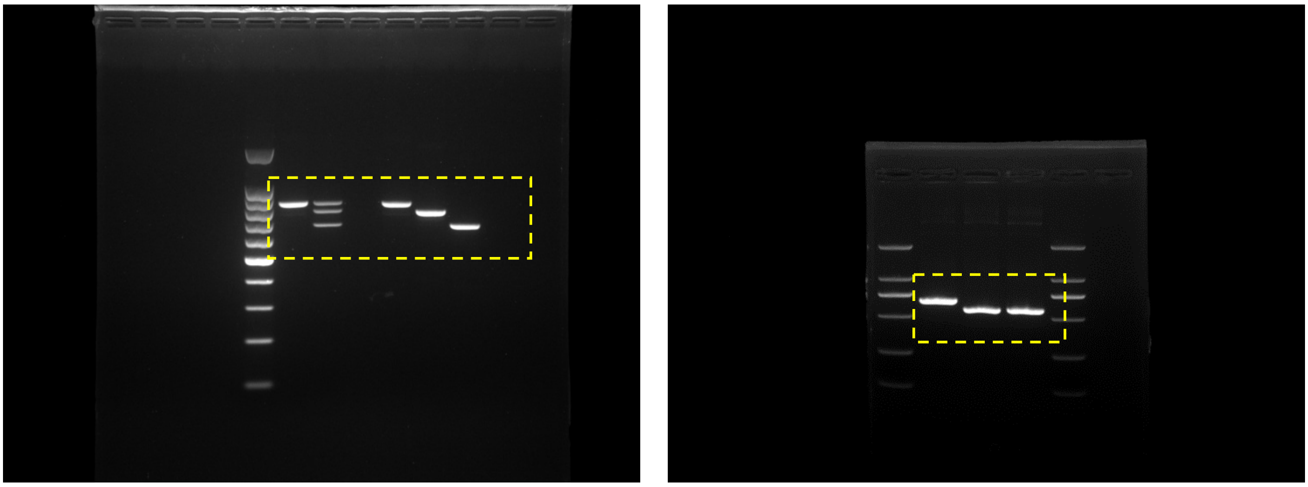

Full unedited blot/gel for Figure 1H

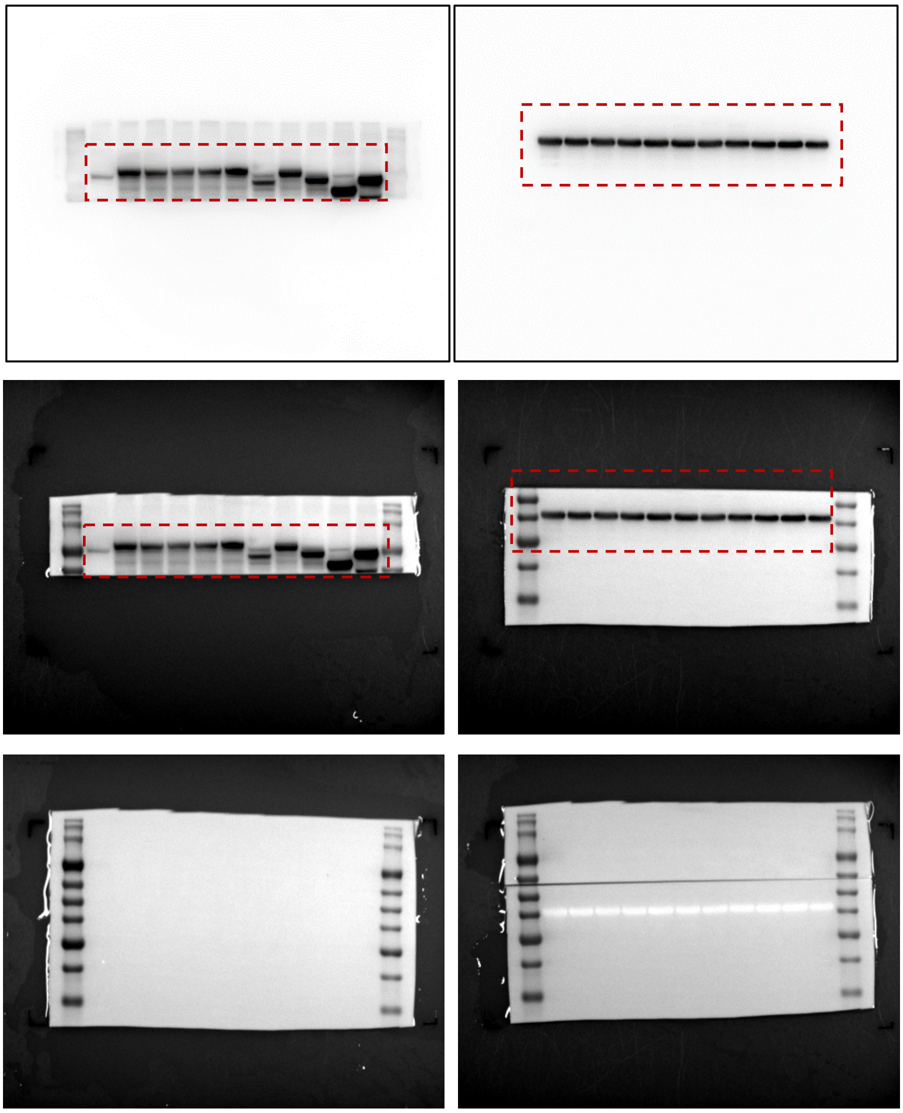

Full unedited blot/gel for Figure 2B-input

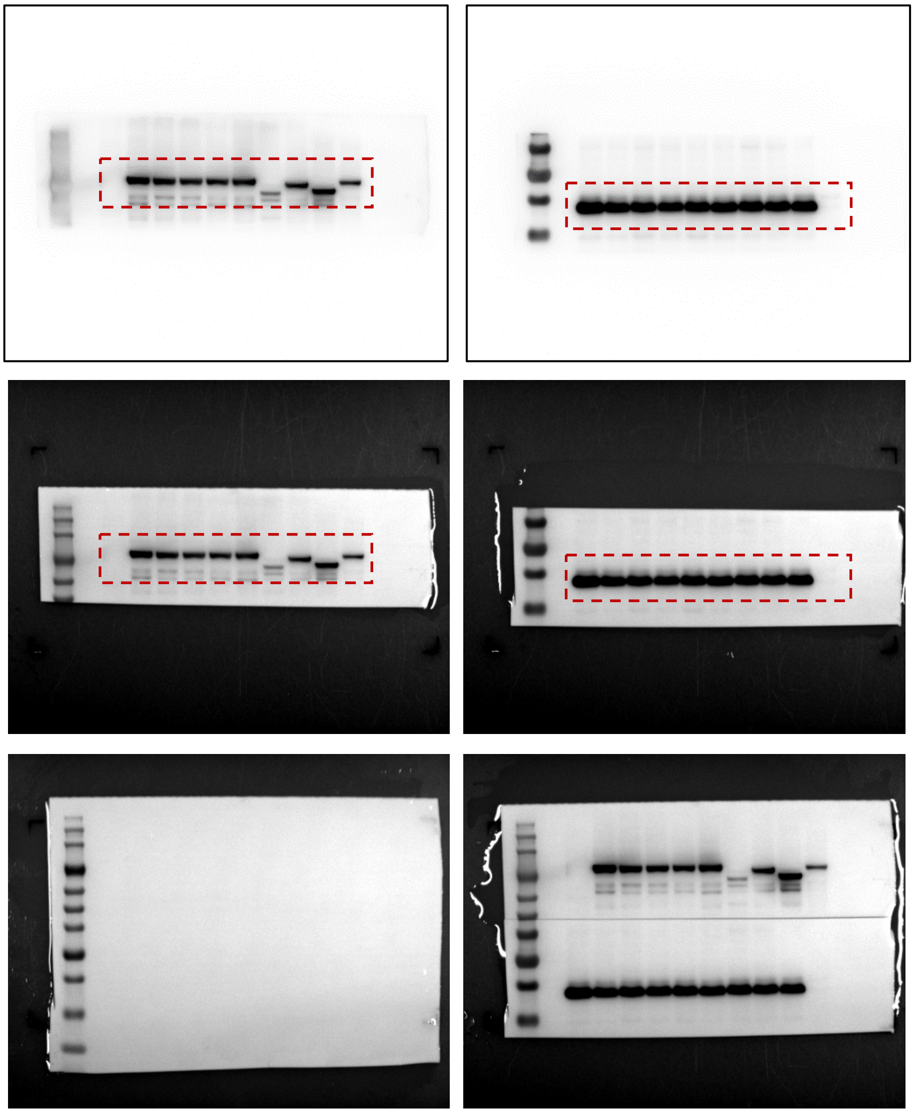

Full unedited blot/gel for Figure 2B-IP-Flag

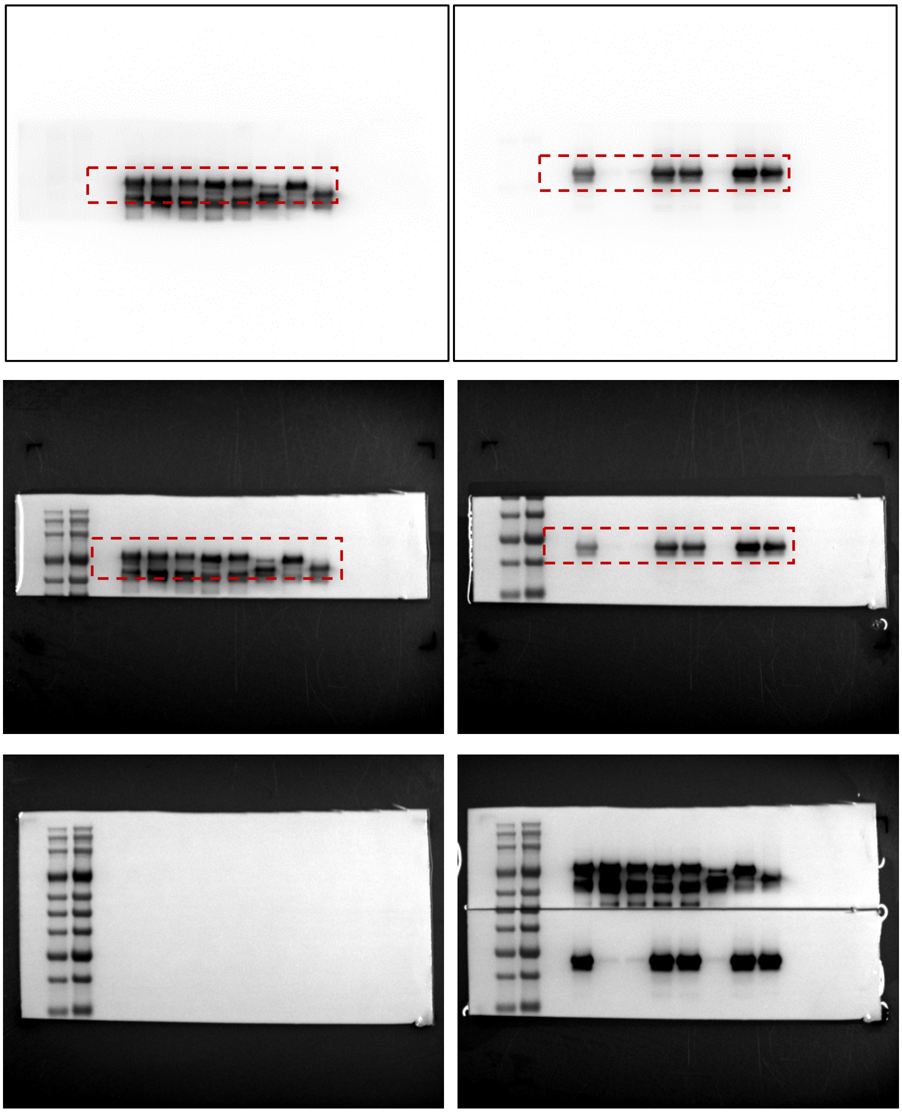

Full unedited blot/gel for Figure 2B-IP-HA

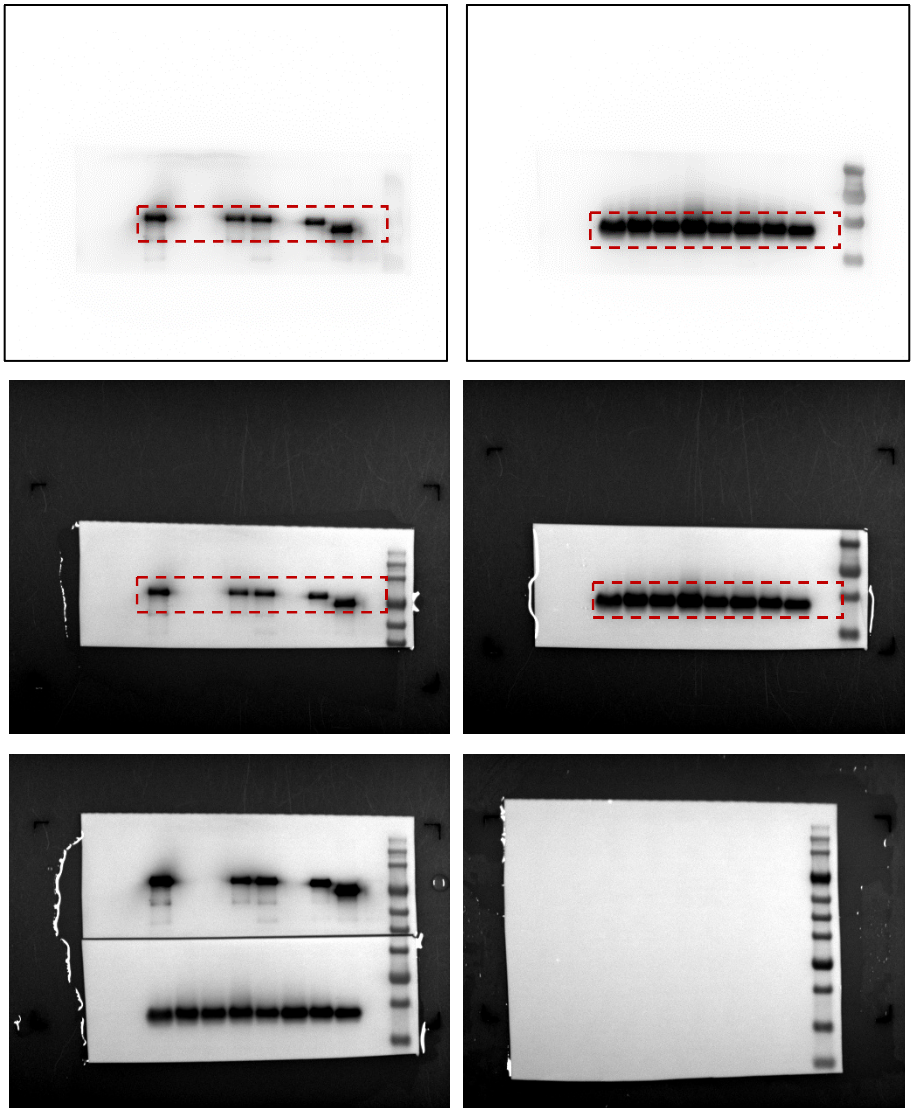

Full unedited blot/gel for Figure 2E-Input

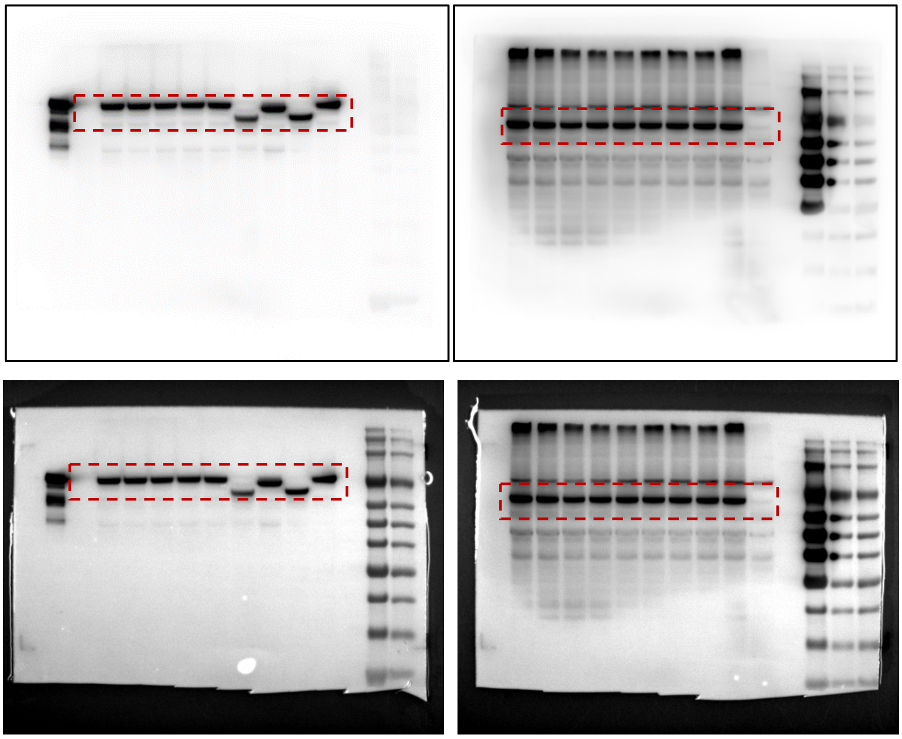

To ensure the accuracy and clarity of our Western blot results, we performed separate blots for each target protein due to their similar molecular weights. When attempting to detect both proteins on the same membrane, stripping and re-probing often resulted in either the loss of the second target band or overexposure caused by residual primary antibody. To avoid these issues, we loaded the same samples under identical conditions onto two separate gels, transferred them to membranes, and probed each membrane with a distinct primary antibody. This approach allowed us to obtain clear and reliable results for both proteins without compromising the integrity of the data.

Full unedited blot/gel for Figure 2E-IP-Flag

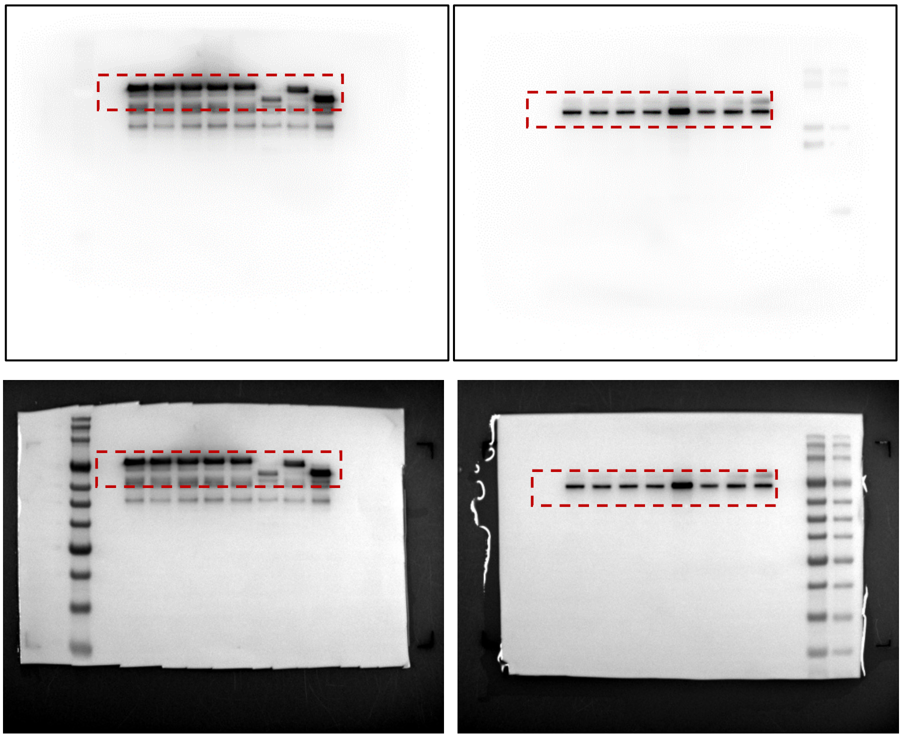

To ensure the accuracy and clarity of our Western blot results, we performed separate blots for each target protein due to their similar molecular weights. When attempting to detect both proteins on the same membrane, stripping and re-probing often resulted in either the loss of the second target band or overexposure caused by residual primary antibody. To avoid these issues, we loaded the same samples under identical conditions onto two separate gels, transferred them to membranes, and probed each membrane with a distinct primary antibody. This approach allowed us to obtain clear and reliable results for both proteins without compromising the integrity of the data.

Full unedited blot/gel for Figure 2E-IP-HA

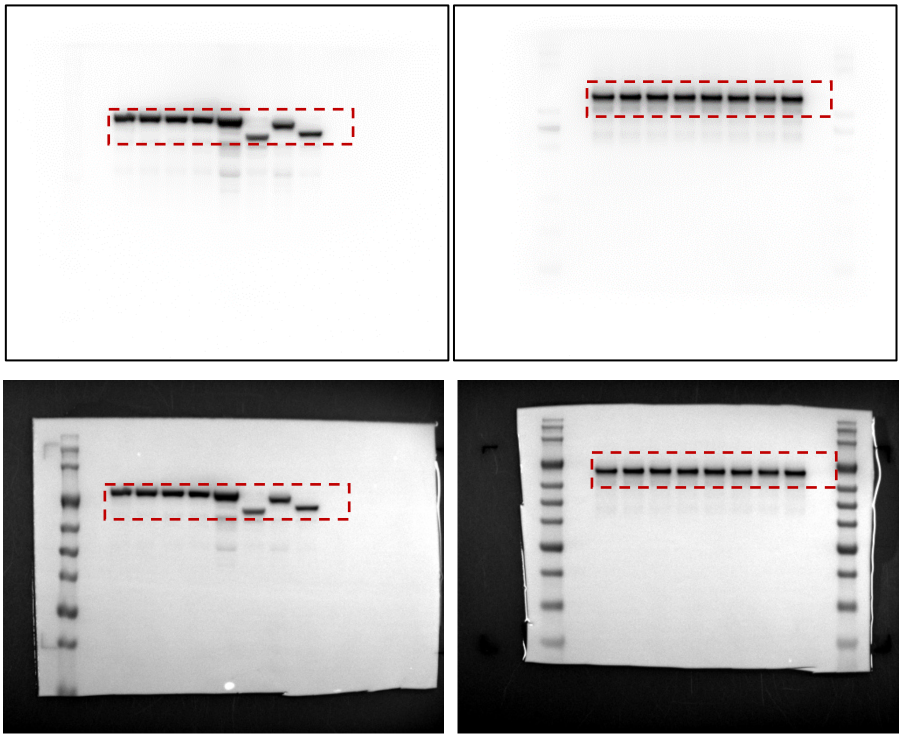

To ensure the accuracy and clarity of our Western blot results, we performed separate blots for each target protein due to their similar molecular weights. When attempting to detect both proteins on the same membrane, stripping and re-probing often resulted in either the loss of the second target band or overexposure caused by residual primary antibody. To avoid these issues, we loaded the same samples under identical conditions onto two separate gels, transferred them to membranes, and probed each membrane with a distinct primary antibody. This approach allowed us to obtain clear and reliable results for both proteins without compromising the integrity of the data.

Full unedited blot/gel for Figure 2G-Input

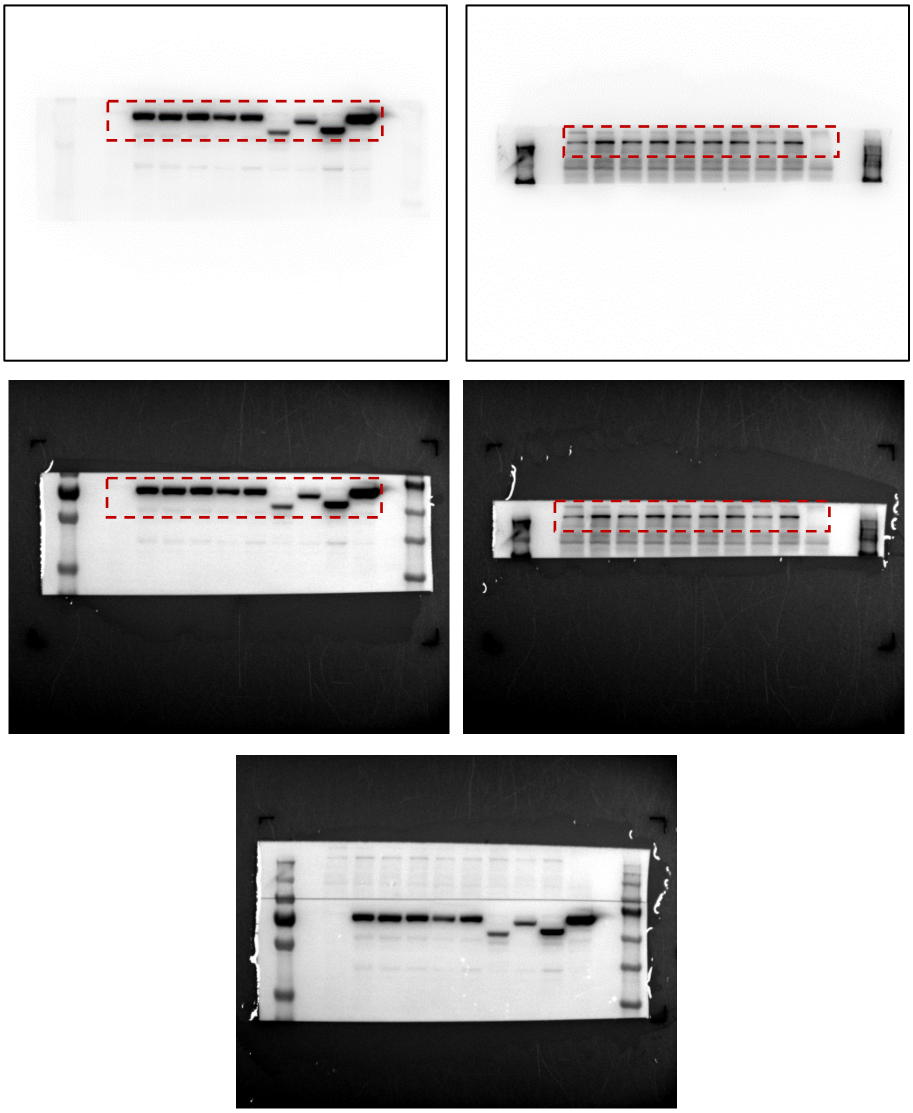

Full unedited blot/gel for Figure 2G-IP-Flag

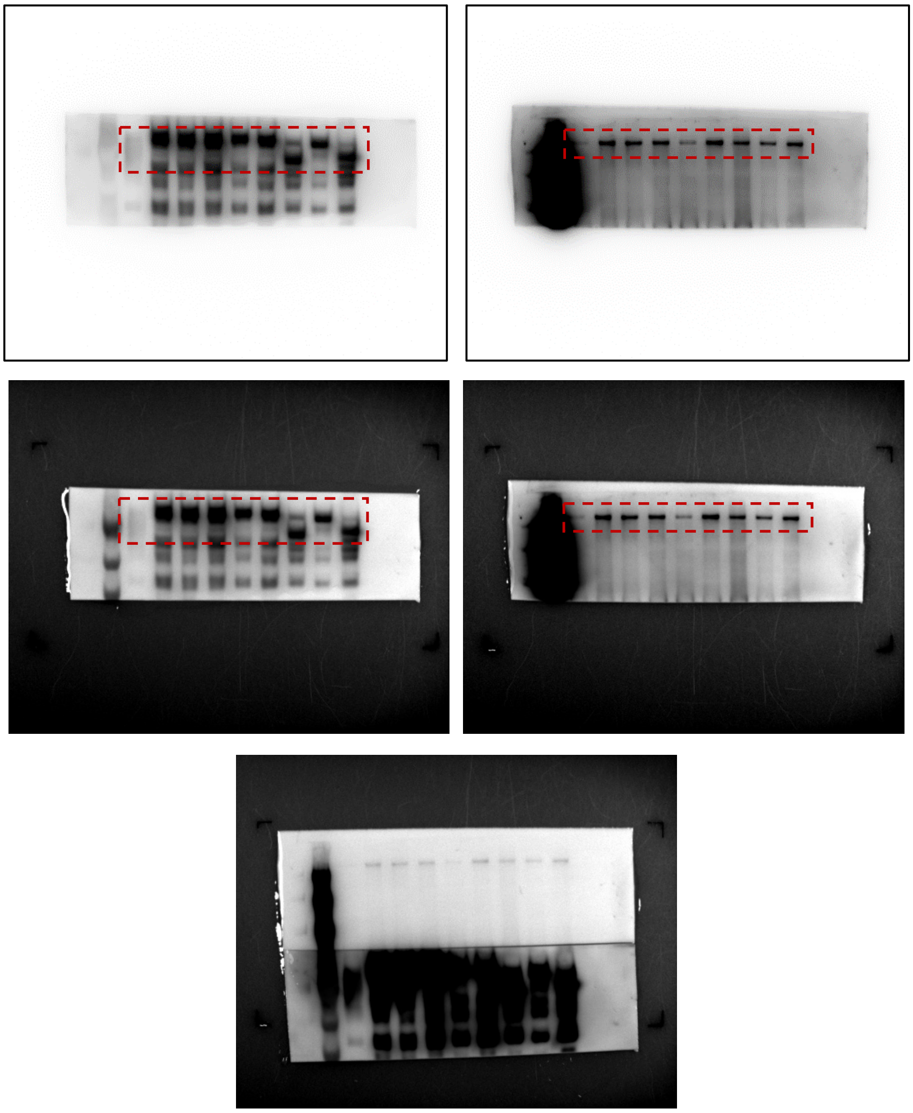

Full unedited blot/gel for Figure 2G-IP-HA

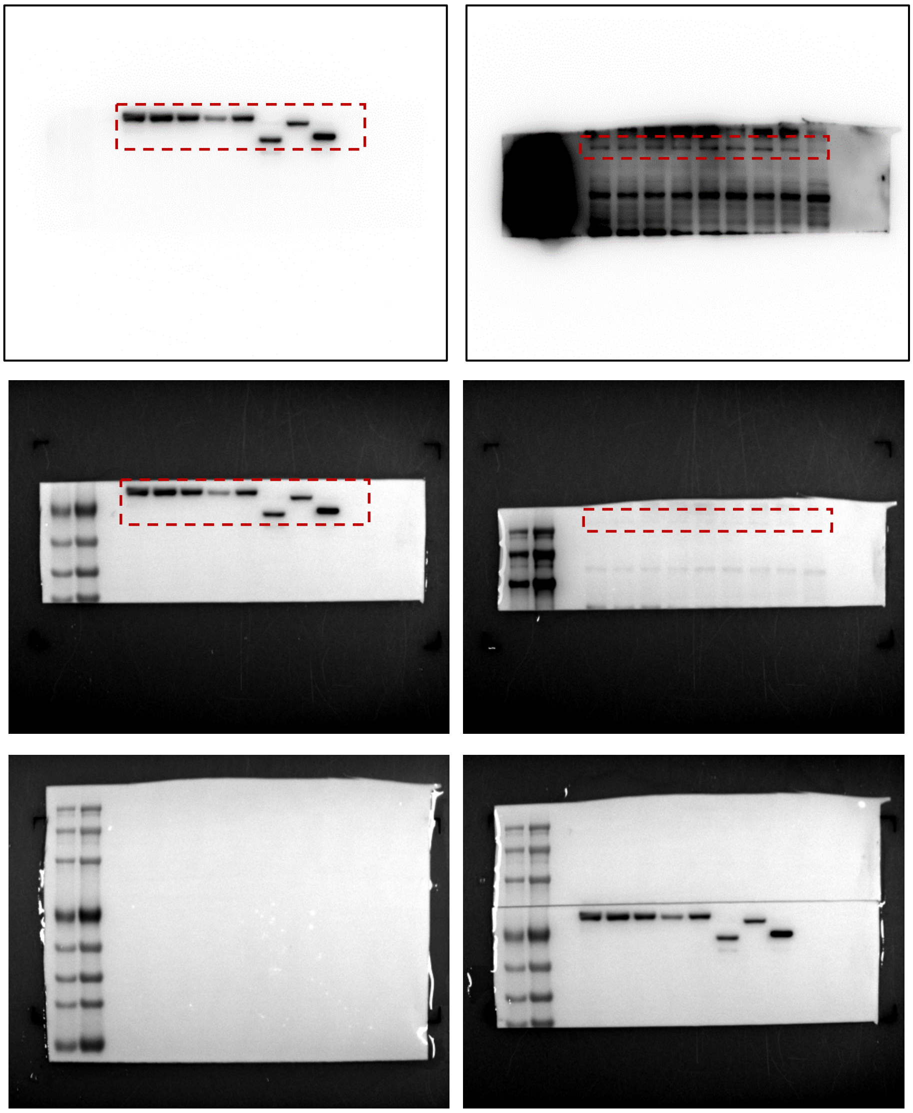

Full unedited blot/gel for Supplementary Figure 3D

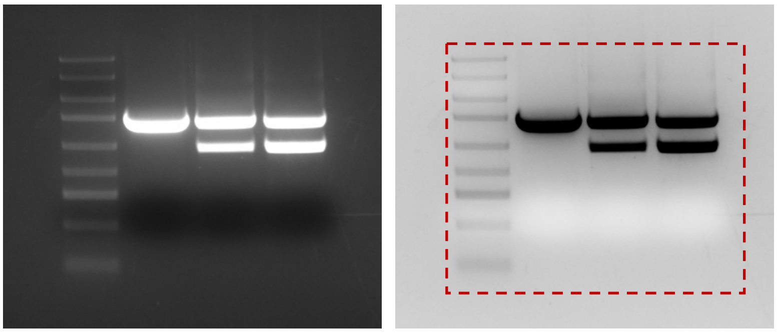

Full unedited blot/gel for Supplementary Figure 3H

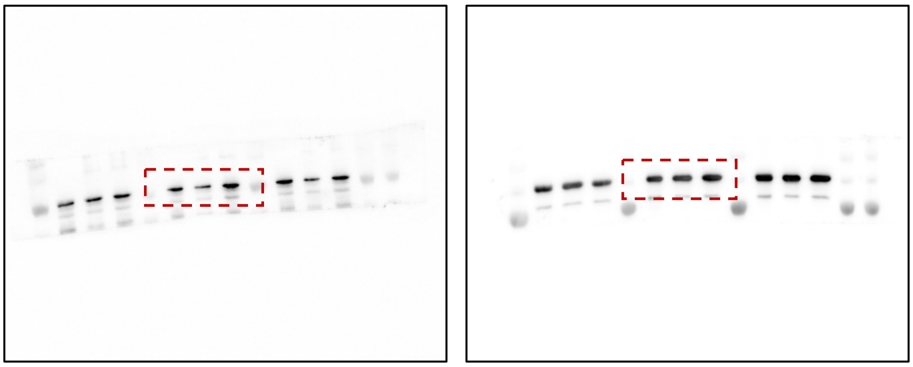

Full unedited blot/gel for Supplementary Figure 1F
